# Degradation of STIM1 through FAM134B-mediated ER-phagy is potentially involved in cell proliferation

**DOI:** 10.1016/j.jbc.2024.107674

**Published:** 2024-08-14

**Authors:** Hiroaki Kajiho, Toshiaki Sakisaka

**Affiliations:** 1 Division of Membrane Dynamics, Department of Physiology and Cell Biology, Kobe University Graduate School of Medicine, Kobe, Japan; 2 Department of Biochemical Pathophysiology, Medical Research Institute, Tokyo Medical and Dental University, Tokyo, Japan

**Keywords:** autophagy, cell cycle, cell proliferation, ER-phagy, endoplasmic reticulum (ER), FAM134B, protein degradation, STIM1

## Abstract

Autophagy is classified as nonselective or selective depending on the types of degrading substrates. Endoplasmic reticulum (ER)-phagy is a form of selective autophagy for transporting the ER-resident proteins to autolysosomes. FAM134B, a member of the family with sequence similarity 134, is a well-known ER-phagy receptor. Dysfunction of FAM134B results in several diseases including viral infection, inflammation, neurodegenerative disorder, and cancer, indicating that FAM134B has crucial roles in various kinds of intracellular functions. However, how FAM134B-mediated ER-phagy regulates intracellular functions is not well understood. In this study, we found that FAM134B knockdown in mammalian cells accelerated cell proliferation. FAM134B knockdown increased the protein amount of stromal interaction molecule 1 (STIM1), an ER Ca^2+^ sensor protein mediating the store-operated Ca^2+^ entry involved in G1 to S phase transition. FAM134B bound to STIM1 through its C-terminal cytosolic region. FAM134B knockdown reduced transport of STIM1 from the ER to autolysosomes. Finally, FAM134B knockdown accelerated G1 to S phase transition. These results suggest that FAM134B is involved in cell proliferation possibly through degradation of STIM1 *via* ER-phagy.

Cells degrade intracellular components to regenerate proteins and lipids and to produce energy for survival under starved conditions. This self-digestion process is called autophagy. Microtubule-associated protein light chain 3 (LC3) is a soluble protein. In starved conditions, a cytosolic form of LC3 (LC3-I) is conjugated to phosphatidylethanolamine (PE) to form LC3-PE (LC3-II). LC3-II anchors to nascent membranes introduced by starved signals (1). LC3-II-anchored membranes, called as isolation membranes, wrap part of intracellular components to form autophagosomes. Autophagosomes undergo a maturation process by fusing with lysosomes to form autolysosomes, where the intracellular components are digested by lysosomal hydrolases. Autophagy has been considered as a nonselective pathway for engulfing and degrading cytoplasm (1, 2). Recently, several studies have demonstrated that selective autophagy, another mode of autophagy, is involved in clearance of specific organelles, proteins, and pathogens (3, 4). Selective autophagy is achieved by the interaction of specific receptors localizing at engulfed components with LC3 on isolation membranes.

Endoplasmic reticulum (ER)-phagy is a form of selective autophagy for the ER-resident proteins, and is mediated by ER-phagy receptors. Mammalian ER-phagy receptors comprise family with sequence similarity 134 member B (FAM134B), cell cycle progression 1 (CCPG1), translocation protein SEC62, testis expressed 264 (TEX264), atlastin 3 (ATL3) and the long isoform of reticulon 3 (RTN3L) (5). All the ER-phagy receptors share a common motif called LC3 interacting regions (LIRs). Through their LIRs, the ER-phagy receptors on the ER membrane bind to LC3 during ER-phagy. Among the ER-phagy receptors, FAM134B has been well-characterized. FAM134B was originally identified as an oncogene found in esophageal squamous cell carcinoma, and dysfunction of FAM134B results in viral infection, inflammation, hereditary sensory and autonomic neuropathy type II, and cancer (6, 7, 8, 9, 10). In addition, degeneration of sensory neurons was observed in *Fam134b* KO mice (11), indicating that FAM134B has crucial roles in various kinds of intracellular functions. FAM134B is composed of a short N-terminal cytosolic region, reticulon-homology domain (RHD) and a long C-terminal cytosolic region containing LIR. Similar to other RHD-containing proteins including reticulon (RTN) family, REEPs/DP1/Yop1 and Arl6IP1, FAM134B forms oligomers through its RHD. A study has shown that phosphorylation of FAM134B at serine 151 by Ca^2+^/calmodulin-dependent protein kinase II beta (CAMK2B) promotes oligomerization (12). Oligomerized FAM134B binds to Arl6IP1 and generates high membrane curvature of the ER, thereby preparing for isolation by phagophore (13), suggesting that intracellular Ca^2+^ concentration affects ER-phagy through FAM134B oligomerization.

ER-phagy is active not only under stress conditions but also under basal conditions (14, 15). The ER is composed of a dynamic network of interconnected sheets and tubules in interphase. The ER constitutively undergoes constant remodeling through elongation and retraction of tubules (16). ER-phagy is involved in ER remodeling to preserve the size and morphology of the ER (17). Not only in interphase, the ER is also dramatically remodeled in mitosis. The ER is fenestrated in anaphase and divided equally into two daughter cells (18, 19). The fenestrated ER is converted into the ER sheets and tubules in G1 phase, raising the possibility that ER-phagy would be active in G1 phase.

In G1 phase, cells repair the damaged DNA during replication in the previous cell cycle, and store enough materials for DNA synthesis in next S phase. Therefore, G1 to S phase transition is a checkpoint where the completion of G1 phase has to be strictly verified for the proper cell proliferation. Recent studies have shown that stromal interaction molecule 1 (STIM1), a Ca^2+^ sensor in the ER tubules, controls G1 to S phase transition (20, 21, 22). STIM1 binds to Orai1 on the plasma membrane, and the STIM1-Orai1 complex mediates the store-operated Ca^2+^ entry (SOCE) from extracellular milieu (23, 24). STIM1 knockdown blocks SOCE and arrests cell cycles at G1 to S phase transition, indicating that regulation of the protein amount of STIM1 is important for G1 to S phase transition. However, little is known about how degradation of STIM1 is regulated except for the reports showing that STIM1 is ubiquitinated by Plenty of SH3’s and tripartite motif containing 32 and that mitotic STIM1 is targeted to autophagy pathway (25, 26, 27).

In this study, we found that FAM134B knockdown promoted cell proliferation and increased the protein amount of STIM1. FAM134B bound to STIM1 through its C-terminal cytosolic region. FAM134B knockdown reduced transport of STIM1 from the ER to autolysosomes and accelerated G1 to S phase transition. Our findings underscore that FAM134B is involved in G1 to S phase transition possibly through degradation of STIM1, and imply a novel role of ER-phagy in the regulation of cell proliferation.

## Results

### FAM134B knockdown promotes cell proliferation

**Figure 1:**
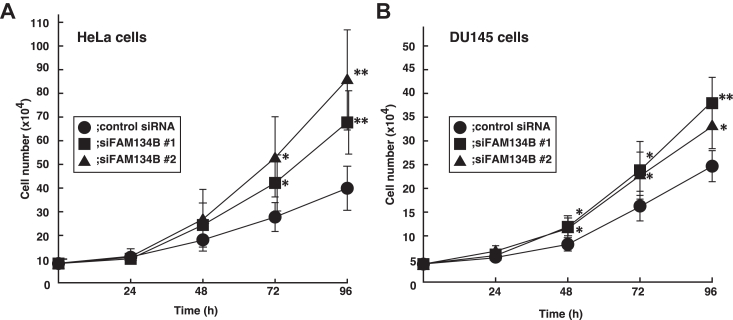
**FAM134B knockdown promotes cell proliferation.** The control siRNA (*circles*) or two independent siRNAs targeting FAM134B, siFAM134B #1 (*squares*), or siFAM134B #2 (*triangles*) was transfected into HeLa (*A*) or DU145 (*B*) cells with at a density of 80,000 or 40,000 cells, respectively. Briefly, 24, 48, 72, and 96 h after transfection, the cell numbers were counted. Data are the averages ± SD of three independent experiments. ∗*p* < 0.05, ∗∗*p* < 0.01 *versus* the control siRNA-transfected cells in each time point by paired Student’s *t* test. FAM134B, family with sequence similarity 134 member B.

To understand the functions of FAM134B, we performed experiments in which siRNAs targeting FAM134B (siFAM134B) were transfected into HeLa cells. Unexpectedly, the siFAM134B-transfected cells appeared to be smaller and grow faster than the control-siRNA transfected cells. Therefore, we analyzed whether FAM134B knockdown affects cell proliferation. FAM134B has two splicing forms, a long isoform (FAM134B-1) and an N-terminal truncated isoform (FAM134B-2) (28). We examined the expression levels of endogenous FAM134B in several cell lines by immunoblotting. HeLa cells expressed both FAM134B-1 and FAM134B-2 (Fig. S1). In addition, DU145 cells expressed relatively high amount of endogenous FAM134B-1 among the cell lines we tested. Therefore, we performed cell proliferation assay using HeLa and DU145 cells. The control siRNA or two independent siFAM134Bs (siFAM134B #1 and #2) was transfected into HeLa or DU145 cells. Briefly, 24, 48, 72, and 96 h after transfection, the number of the viable cells was counted by using trypan blue exclusion method. In both HeLa and DU145 cells, the number of siFAM134B #1 or #2-transfected cells increased by more than 60% or 40% compared to that of the control siRNA-transfected cells, respectively (Fig. 1).

Collectively, these results indicate that FAM134B knockdown promotes cell proliferation.

### FAM134B is involved in the degradation of STIM1

**Figure 2:**
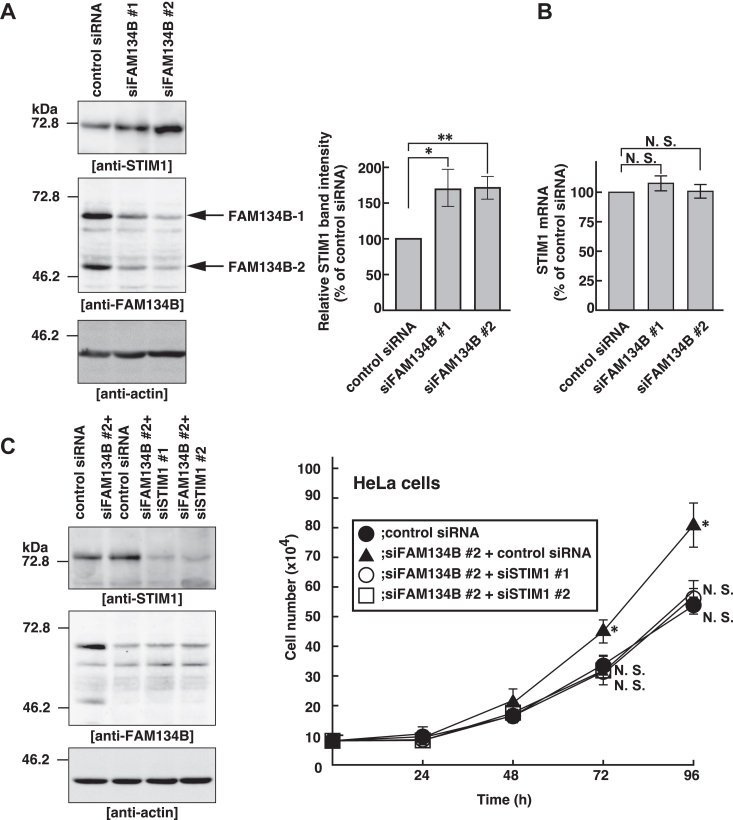
**FAM134B is involved in the degradation of STIM1.***A*, increase in the protein amount of endogenous STIM1 in the FAM134B knockdown cells. The control siRNA, siFAM134B #1, or siFAM134B #2 was transfected into HeLa cells. 15 μg of whole cell lysates were subjected to SDS-PAGE followed by immunoblotting with the anti-STIM1 pAb, the anti-FAM134B pAb and the anti-actin mAb (*left* panel). The intensities of immunoreactive bands for STIM1 were normalized to those for actin and expressed as percentages of the control siRNA-transfected cells (*right* panel). Data are the averages ± SD of three independent experiments. ∗*p* < 0.05, ∗∗*p* < 0.01 *versus* the control siRNA-transfected cells by paired Student’s *t* test. *B*, no significant increase in the amount of STIM1 mRNA in the FAM134B knockdown cells. The amount of STIM1 mRNA in the cells was quantified by quantitative PCR. They were normalized to those of GAPDH mRNA, and expressed as percentages of the control siRNA-transfected cells. Data are the averages ± SD of three independent experiments. N. S.: not significant *versus* the control siRNA-transfected cells by paired Student’s *t* test. *C*, restoration of cell proliferation in the FAM134B knockdown cells by STIM1 knockdown. The control siRNA (*closed circles*), siFAM134B #2+the control siRNA (*closed triangles*), siFAM134B #2+siSTIM1 #1 (*open circles*), or siFAM134B #2+siSTIM1 #2 (*open squares*) was transfected into HeLa cells with at a density of 80,000 cells. In addition, 15 μg of whole cell lysates were subjected to SDS-PAGE followed by immunoblotting with the anti-STIM1 pAb, the anti-FAM134B pAb, and the anti-actin mAb (*left* panel). Briefly, 24, 48, 72, and 96 h after transfection, the cell numbers were counted (*right* panel). Data are the averages ± SD of three independent experiments. ∗*p* < 0.05, N. S.: not significant *versus* the control siRNA-transfected cells by paired Student’s *t* test. FAM134B, family with sequence similarity 134 member B; STIM1, stromal interaction molecule 1.

FAM134B is well-known as an ER-phagy receptor and mediates the lysosomal degradation of the specific ER-localizing proteins (11). STIM1 is an ER Ca^2+^ sensor protein that binds to a membrane Ca^2+^ channel Orai1, and the STIM1-Orai1 complex induces Ca^2+^ influx by SOCE (23, 24). Recent studies have shown that STIM1 knockdown reduces cell proliferation due to the delay of G1 to S phase transition of the cell cycle (20, 21, 22). Therefore, we reasoned that FAM134B knockdown might promote cell proliferation by increasing the protein amount of STIM1. To examine this reasoning, the control siRNA, siFAM134B #1 or #2 was transfected into HeLa cells and the cell lysates were subjected to SDS-PAGE followed by immunoblotting with the anti-STIM1 pAb, the anti-FAM134B pAb, and the anti-actin mAb. siFAM134B #1 and #2 successfully knocked down both endogenous FAM134B-1 and FAM134B-2 as confirmed by immunoblotting with the anti-FAM134B pAb (Fig. 2*A*, middle panel). The protein amount of endogenous STIM1 was significantly increased in the siFAM134B #1 or #2-transfected cells relative to the control siRNA-transfected cells (Fig. 2*A*, upper panel). To rule out the possibility that the increase of the protein amount of STIM1 in the siFAM134B-transfected cells is attributed to the increased transcription of STIM1 mRNA, STIM1 mRNA in the control-siRNA, or siFAM134B #1 or #2-transfected cells were quantified by quantitative PCR. The mRNA amount of STIM1 was not increased in the siFAM134B #1 or #2-transfected cells (Fig. 2*B*), suggesting that FAM134B knockdown reduces the degradation of STIM1 protein.

We reasoned if promotion of cell proliferation of the FAM134B knockdown cells is attributed to increased amount of STIM1, knockdown of STIM1 would reverse promotion of cell proliferation of the FAM134B knockdown cells. The control siRNA or two independent siRNAs targeting STIM1 (siSTIM1 #1 and siSTIM1 #2) along with siFAM134B #2 was cotransfected into HeLa cells. Subsequently, 24, 48, 72, and 96 h after transfection, the cell numbers were counted. The protein amount of endogenous STIM1 was increased in the siFAM134B #2-transfected cells, and the increased protein amount of STIM1 was successfully knocked down by siSTIM1 #1 and #2, as confirmed by immunoblotting with the anti-STIM1 pAb (Fig. 2*C*, left panel). siSTIM1 #1 or siSTIM1 #2 but not the control siRNA reversed promotion of cell proliferation of the siFAM134B #2-transfected cells (Fig. 2*C*, right panel), which supports our conclusion that promotion of cell proliferation of the FAM134B knockdown cells is attributed to increased amount of STIM1.

The increased amount of STIM1 by FAM134B knockdown might cause ER stress. Therefore, we examined the expression of immunoglobulin heavy-chain binding protein (BiP), widely used as a marker for ER stress (29). HeLa cells were transfected with the control siRNA, siFAM134B #1 or siFAM134B #2. As a positive control for ER stress, HeLa cells were cultured in the absence or presence of thapsigargin, an ER stress inducer by inhibiting the ER Ca^2+^-ATPase. Whole cell lysates of the cells were immunoblotted with the anti-BiP pAb, the anti-STIM1 pAb, the anti-FAM134B pAb, and the anti-actin mAb. As expected, the protein amount of BiP was increased by thapsigargin treatment (Fig. S2). In contrast, the protein amount of BiP in the siFAM134B #1 or #2-transfected cells was comparable to those in the control siRNA-transfected cells. These results indicate that the increased amount of STIM1 by FAM134B knockdown apparently does not cause ER stress.

These results suggest that FAM134B is involved in the degradation of STIM1, and that the increased amount of STIM1 promotes cell proliferation.

### FAM134B binds to STIM1 through its C-terminal cytosolic region

**Figure 3:**
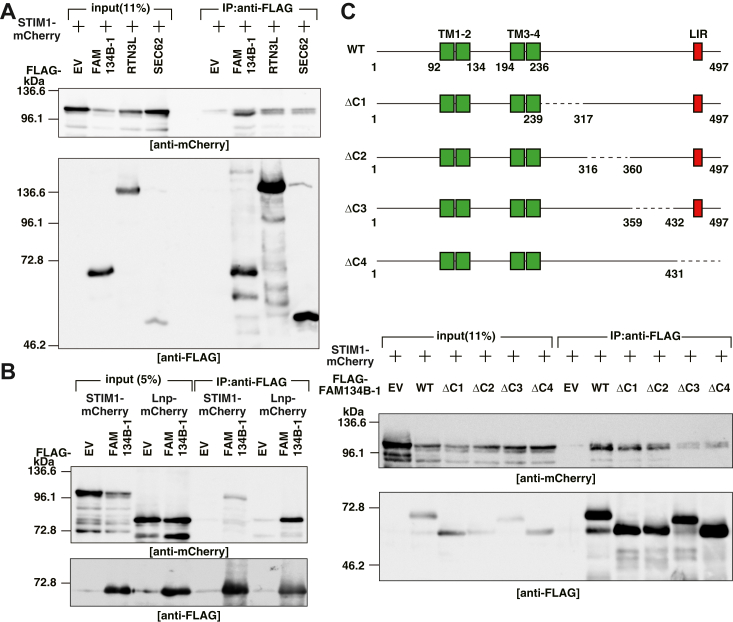
**FAM134B binds to STIM1 through its C-terminal cytosolic region.***A*, coimmunoprecipitation of the ER-phagy receptors and STIM1. FLAG-EV, FAM134B-1, RTN3L, or SEC62 was cotransfected into HEK293 cells along with STIM1-mCherry. NP-40 extracts of the transfected cells were immunoprecipitated with the anti-FLAG mAb, followed by immunoblotting with the anti-mCherry pAb and the anti-FLAG pAb. *B*, reduction of the protein amount of STIM1 by the expression of FAM134B-1. FLAG-EV or FAM134B-1 was cotransfected with STIM1- or Lnp-mCherry into HEK293 cells. NP-40 extracts of the transfected cells were immunoprecipitated with the anti-FLAG mAb. The immunoprecipitated and the input samples were immunoblotted with the anti-mCherry pAb and the anti-FLAG pAb. *C*, coimmunoprecipitation of the deletion mutants of FAM134B-1 and STIM1. The primary structures of FAM134B-1 and the deletion mutants are represented (*upper* panel). TM, transmembrane; LIR, LC3 interacting region. FLAG-EV, FAM134B-1 WT, ΔC1, ΔC2, ΔC3, or ΔC4 was cotransfected into HEK293 cells along with STIM1-mCherry. NP-40 extracts of the transfected cells were immunoprecipitated with the anti-FLAG mAb. The immunoprecipitated and the input samples were immunoblotted with the anti-mCherry pAb and the anti-FLAG pAb (*middle* and *lower* panels). ER, endoplasmic reticulum; EV, empty vector; FAM134B, family with sequence similarity 134 member B; LC3, light chain 3; Lnp, lunapark; RTN3L, long isoform of reticulon 3; STIM1, stromal interaction molecule 1.

We examined whether FAM134B binds to STIM1 by immunoprecipitation analysis. ER-phagy takes place both at the ER tubules and sheets. Therefore, we also examined other ER-phagy receptors, RTN3L and SEC62 which localize at the ER tubules and sheets, respectively, bind to STIM1. FLAG-tagged empty vector (EV), FAM134B-1, RTN3L, or SEC62 (FLAG-EV, FAM134B-1, RTN3L, or SEC62, respectively) was transfected into HEK293 cells along with C-terminal mCherry-tagged STIM1 (STIM1-mCherry), followed by immunoprecipitation with the anti-FLAG mAb. The samples were subjected to SDS-PAGE followed by immunoblotting with the anti-mCherry pAb and the anti-FLAG pAb. FLAG-FAM134B-1, RTN3L, and SEC62 were immunoprecipitated by the anti-FLAG mAb (Fig. 3*A*, lower panel), and STIM1-mCherry was coimmunoprecipitated with FLAG-FAM134B-1 more efficiently than FLAG-RTN3L and SEC62 (Fig. 3*A*, upper panel). Of note, the protein amount of STIM1-mCherry (shown as “input”) was reduced by the expression of FLAG-FAM134B-1 relative to FLAG-EV, RTN3L, or SEC62 (Fig. 3*A*, upper panel). The reduction of STIM1-mCherry in the FLAG-FAM134B-1 expressing cells is consistent with the result that endogenous STIM1 was increased in the FAM134B knockdown cells (Fig. 2*A*). To rule out the possibility that the reduction of STIM1 in the FAM134B-1 expressing cells is attributed to the nonselective degradation of the ER-membrane proteins by FLAG-FAM134B-1, we examined the protein amount of an ER membrane protein, lunapark (Lnp), in the FAM134B-1 expressing cells. Lnp is important for stabilization of the ER tubular network (30). C-terminal mCherry-tagged STIM1 or Lnp (STIM1-or Lnp-mCherry) was cotransfected with FLAG-EV or FAM134B-1 into HEK293 cells, followed by immunoprecipitation with the anti-FLAG mAb. The immunoprecipitated and input samples were subjected to SDS-PAGE followed by immunoblotting with the anti-mCherry pAb and the anti-FLAG pAb. FLAG-FAM134B-1 was immunoprecipitated by the anti-FLAG mAb (Fig. 3*B*, lower panel), and STIM1-and Lnp-mCherry were coimmunoprecipitated with FLAG-FAM134B-1 (Fig. 3*B*, upper panel), indicating that STIM1 and Lnp bind to FAM134B-1. Immunoblot analysis of the input samples showed that the protein amount of STIM1-mCherry was reduced by the expression of FLAG-FAM134B-1 (Fig. 3*B*, upper panel). In contrast, the protein amount of Lnp-mCherry was not reduced by the expression of FLAG-FAM134B-1. These results suggest that STIM1 was specifically degraded by FAM134B irrespective of the binding potency to FAM134B.

To define the STIM1-binding region of FAM134B-1, a set of mutants truncated at the C-terminal cytosolic region of FAM134B-1 (ΔC1, ΔC2, ΔC3, and ΔC4) were generated (Fig. 3*C*, upper panel). WT or the mutants of FLAG-FAM134B-1 was cotransfected with STIM1-mCherry into HEK293 cells, followed by immunoprecipitation with the anti-FLAG mAb. The samples were subjected to SDS-PAGE followed by immunoblotting with the anti-mCherry pAb and the anti-FLAG pAb. WT and the deletion mutants of FLAG-FAM134B-1 were immunoprecipitated by the anti-FLAG mAb (Fig. 3*C*, lower panel). STIM1-mCherry was coimmunoprecipitated with FLAG-FAM134B-1 WT, ΔC1, and ΔC2, whereas binding of STIM1-mCherry to FLAG-FAM134B-1 ΔC3 and ΔC4 were severely decreased relative to WT, ΔC1, and ΔC2 (Fig. 3*C*, middle panel). The protein amount of STIM1-mCherry was decreased in the FLAG-FAM134B-1 WT, ΔC1, or ΔC2 expressing cells, whereas the protein amount of STIM1-mCherry was not decreased in the FLAG-FAM134B-1 ΔC3 or ΔC4 expressing cells (Fig. 3*C*, middle panel), further supporting our idea that FAM134B binds to STIM1 for degradation by ER-phagy. We also examined whether Lnp-mCherry binds to the mutants of FLAG-FAM134B-1. WT and the deletion mutants of FLAG-FAM134B-1 were immunoprecipitated by the anti-FLAG mAb (Fig. S3, lower panel). Lnp-mCherry was coimmunoprecipitated with FLAG-FAM134B-1 WT and all the deletion mutants, and the protein amount of Lnp-mCherry was comparable among all the samples (Fig. S3, upper panel).

We further defined the FAM134B-binding region in STIM1. STIM1 comprises three coiled-coiled domains (CC1, 2 and 3) in the C-terminal cytosolic region (23). CC2 and CC3 in STIM1 are STIM1 Orai1 activating region (SOAR), by which STIM1 binds to Orai1 (23, 31). A set of mutants truncated at the C-terminal cytosolic region of STIM1 (ΔC1 and ΔC2) were generated (Fig. S4, upper panel). WT or the mutants of STIM1-mCherry was cotransfected with FLAG-FAM134B-1 into HEK293 cells, followed by immunoprecipitation with the anti-FLAG mAb. The samples were subjected to SDS-PAGE followed by immunoblotting with the anti-mCherry pAb and the anti-FLAG pAb. FLAG-FAM134B-1 was immunoprecipitated by the anti-FLAG mAb (Fig. S4, lower panel), and STIM1 WT, ΔC1, and ΔC2-mCherry were coimmunoprecipitated with FLAG-FAM134B-1 (Fig. S4, middle panel). These results suggest that the FAM134B-binding region in STIM1 is different from the Orai1-binding region, SOAR.

Collectively, these results indicate that FAM134B binds to STIM1 through its C-terminal cytosolic region for degradation.

### FAM134B knockdown reduces transport of STIM1 from the ER to autolysosomes

**Figure 4:**
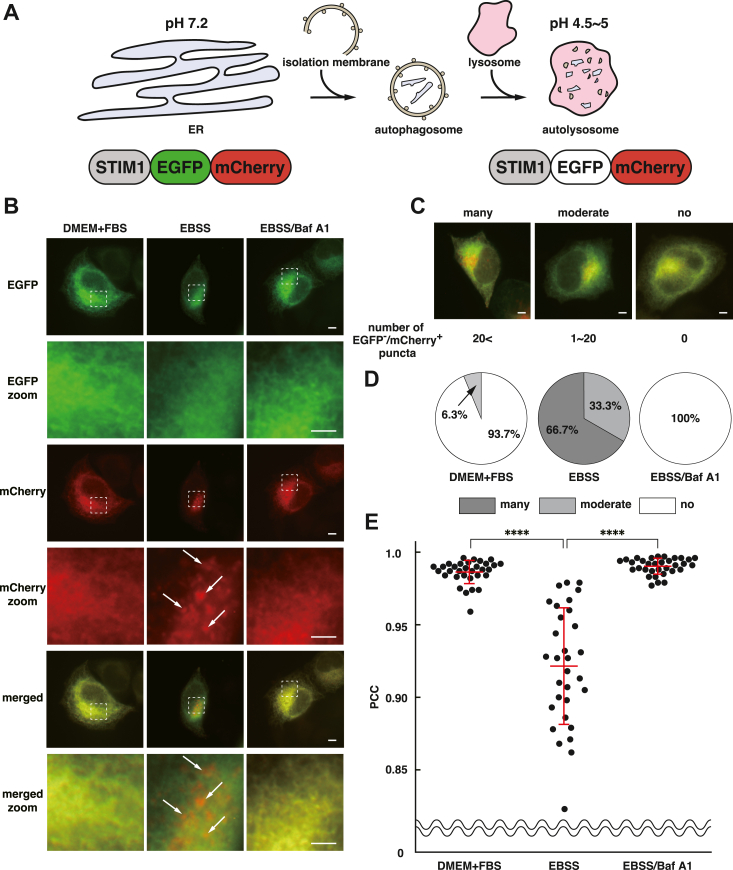
**STIM1-EGFP-mCherry in EATR assay is transported from the ER to autolysosomes.***A*, schematic representation of EATR assay. Under nutrient-rich conditions, the STIM1-EGFP-mCherry reporter emits both *green* and *red* fluorescence at the ER (pH 7.2). Under starved conditions, the ER membrane including the reporter is engulfed by isolation membrane to form autophagosomes. Autophagosomes subsequently fuses with lysosomes to become the autolysosomes in which the reporter emits only *red* fluorescence (pH 4.5∼5). *B*, the reduction of transport of STIM1-EGFP-mCherry from the ER to autolysosomes by Baf A1 treatment. STIM1-EGFP-mCherry was transfected into HeLa cells. Briefly, 24 h after transfection, the cells were cultured in DMEM+FBS or EBSS in the presence of DMSO or 50 nM Baf A1 for 6 h. The *boxed* areas by the *dashed lines* are enlarged to highlight the formation of EGFP^-^/mCherry^+^ puncta in the cells cultured in EBSS but not in DMEM + FBS or EBSS/Baf A1 (zoom). The *arrows* denote representative EGFP^-^/mCherry^+^ puncta. *C*, the representative cell images of the classified categories. Many EGFP^-^/mCherry^+^ puncta (many), a moderate level of EGFP^-^/mCherry^+^ puncta (moderate) and no obvious EGFP^-^/mCherry^+^ punctum (no). Number of EGFP^-^/mCherry^+^ puncta in each category is shown below. *D*, compositions of the categorical data from more than 30 cells in each sample. Many EGFP^-^/mCherry^+^ puncta; *dark gray*, a moderate level of EGFP^-^/mCherry^+^ puncta; *light gray* and no obvious EGFP^-^/mCherry^+^ punctum; *white*. *E*, Pearson’s correlation coefficient (PCC) of EGFP and mCherry signals. Each dot indicates the value of PCC in a single cell. The averages ± SD are indicated by *red lines*. The data were analyzed by one-way ANOVA (F = 85.95, *p* < 0.0001). ∗∗∗∗*p* < 0.0001 *versus* EBSS by Dunnett’s *post hoc* test. The scale bars represent 10 μm (whole cell) and 5 μm (zoom). DMEM, Dulbecco's modified Eagle’s medium; DMSO, dimethylsulfoxide; EATR, ER autophagy tandem reporter; EBSS, Earl's balanced saline solution; EGFP, enhanced green fluorescent protein; ER, endoplasmic reticulum; FBS, fetal bovine serum; STIM1, stromal interaction molecule 1.

As an ER-phagy receptor, FAM134B mediates transport of the ER-resident cargo proteins from the ER to autolysosomes for degradation (11). Therefore, we examined whether FAM134B mediates transport of STIM1 from the ER to autolysosomes by using the ER autophagy tandem reporter (EATR) assay (32). For the assay, we generated a STIM1-enhanced green fluorescent protein (EGFP)-mCherry reporter consisting of STIM1, EGFP, and mCherry (Fig. 4*A*). At neutral pH, both EGFP and mCherry emit fluorescence. In contrast, at low pH, only EGFP is quenched while mCherry continues to emit fluorescence. Therefore, the reporter at the ER (pH 7.2) under nutrient-rich conditions emits both green and red fluorescence, whereas the reporter transported to autolysosomes (pH 4.5∼5) under starved conditions emits only red fluorescence.

First, we examined whether STIM1-EGFP-mCherry is functional as an ER-phagy reporter in EATR assay. STIM1-EGFP-mCherry was transfected into HeLa cells. The cells were cultured for 6 h in Dulbecco's modified Eagle’s medium containing 10% fetal bovine serum (DMEM+FBS) or Earl's balanced saline solution (EBSS) in the presence or absence of 50 nM Baf A1, a selective vacuolar-ATPase inhibitor. In the cells cultured in DMEM+FBS, EGFP and mCherry signals completely colocalized and distributed as a reticular network (Fig. 4*B*). When the cells were starved by EBSS, some EGFP^-^/mCherry^+^ puncta were observed at the perinuclear region of the cells. We categorized the cells into three groups according to the number of EGFP^-^/mCherry^+^ puncta; many EGFP^-^/mCherry^+^ puncta (more than 20 puncta/cell), a moderate level of EGFP^-^/mCherry^+^ puncta (1∼20 puncta/cell) and no obvious EGFP^-^/mCherry^+^ punctum (Fig. 4*C*). 93.7% of the cells cultured in DMEM+FBS formed no obvious EGFP^-^/mCherry^+^ punctum, and 6.3% of the cells formed moderated level of EGFP^-^/mCherry^+^ puncta (Fig. 4*D*). In contrast, 33.3% of the cells cultured in EBSS formed a moderate level of EGFP^-^/mCherry^+^ puncta, and 66.7% of the cells cultured in EBSS formed many EGFP^-^/mCherry^+^ puncta. Baf A1 inhibits vacuolar-ATPase-dependent acidification of lysosomes, resulting in impairment of autophagy influx including ER-phagy influx. Reflecting the inhibitory effect of Baf A1 to autophagy, EGFP^-^/mCherry^+^ puncta completely disappeared in the Baf A1-treated cells cultured in EBSS. To quantitatively analyze the formation of EGFP^-^/mCherry^+^ puncta, Pearson’s correlation coefficient (PCC) of EGFP and mCherry signals was calculated. PCC was significantly reduced from 0.99 to 0.92 by EBSS and was recovered to 0.99 in the ER-phagy-impaired cells by Baf A1 treatment (Fig. 4*E*). These results suggest that STIM1-EGFP-mCherry is transported from the ER to autolysosomes under starved conditions, indicating that the STIM1-EGFP-mCherry is functional as an ER-phagy reporter in EATR assay.

**Figure 5:**
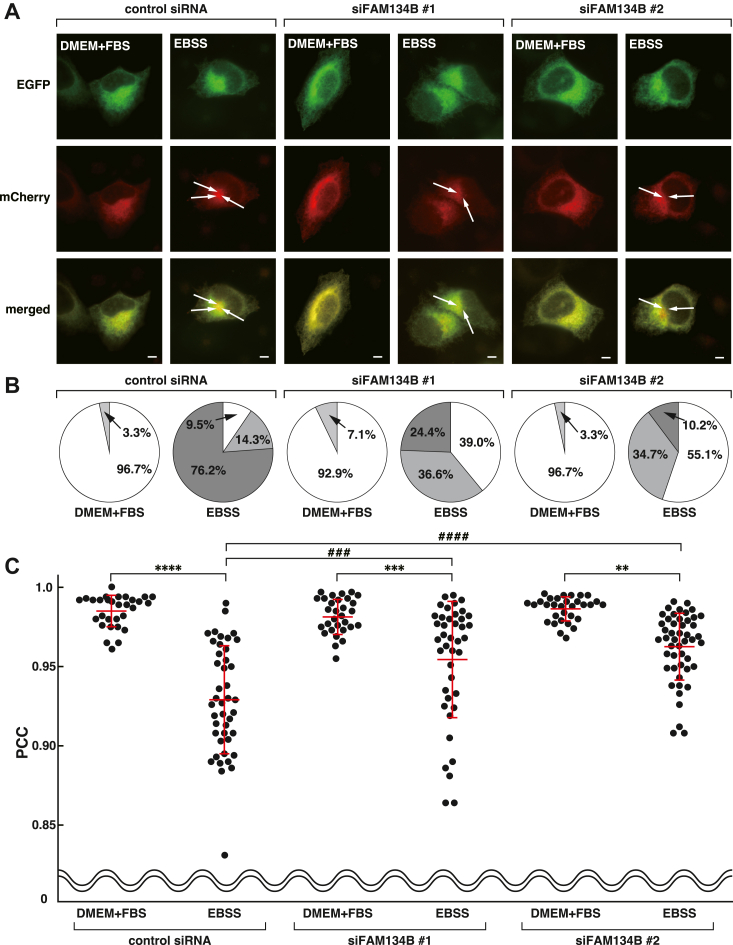
**FAM134B knockdown reduces transport of STIM1 from the ER to autolysosomes.***A*, reduction of transport of STIM1-EGFP-mCherry from the ER to autolysosomes by FAM134B knockdown. HeLa cells were transfected with the control siRNA, siFAM134B #1, or siFAM134B #2, followed by transfection with STIM1-EGFP-mCherry. Twenty-four hours after transfection, the cells were cultured in DMEM+FBS or EBSS for 6 h. The *arrows* denote representative EGFP^-^/mCherry^+^ puncta. *B*, compositions of the categorical data from more than 30 cells in each sample. EGFP^-^/mCherry^+^ puncta formation in the cell was classified into three categories as described in Fig. 4*C*. Many EGFP^-^/mCherry^+^ puncta; *dark gray*, a moderate level of EGFP^-^/mCherry^+^ puncta; *light gray* and no obvious EGFP^-^/mCherry^+^ punctum; *white*. *C*, PCC of EGFP and mCherry signals. Each *dot* indicates the value of PCC in a single cell. The averages ± SD are indicated by *red lines*. The data were analyzed by two-way ANOVA (DMEM+FBS/EBSS: F = 106.6, *p* < 0.0001; siRNAs: F = 10.68, *p* < 0.0001; interaction: F = 10.81, *p* = 0.0002). ∗∗∗∗*p* < 0.0001, ∗∗∗*p* < 0.001, ∗∗*p* < 0.01: DMEM/FBS *versus* EBSS, and ####*p* < 0.0001, ###*p* < 0.001: control siRNA *versus* siFAM134B cultured in EBSS by Tukey’s *post hoc* test. The scale bars represent 10 μm. DMEM, Dulbecco's modified Eagle’s medium; EBSS, Earl's balanced saline solution; EGFP, enhanced green fluorescent protein; ER, endoplasmic reticulum; FAM134B, family with sequence similarity 134 member B; FBS, fetal bovine serum; PCC, Pearson’s correlation coefficient; STIM1, stromal interaction molecule 1.

Next, we examined whether FAM134B knockdown reduces transport of STIM1-EGFP-mCherry from the ER to autolysosomes. STIM1-EGFP-mCherry was transfected into the control siRNA, siFAM134B #1 or #2-transfected HeLa cells. Twenty-four hours after plasmid transfection, the cells were cultured in DMEM+FBS or EBSS for 6 h. Similar to the nontransfected cells shown in Fig. 4*B*, EGFP and mCherry signals completely colocalized and distributed as a reticular network in the control siRNA, siFAM134B #1 or #2-transfected cells cultured in DMEM+FBS (Fig. 5*A*). A total of 76.2% of the control siRNA-transfected cells formed many EGFP^-^/mCherry^+^ puncta when cultured in EBSS (Fig. 5*B*). In contrast, 24.4% or 10.2% of the siFAM134B #1 or #2-transfected cells formed many EGFP^-^/mCherry^+^ puncta, respectively. The average value of PCC of the control siRNA-transfected cells was 0.93, whereas those of the siFAM134B #1 or #2-transfected cells significantly increased up to 0.95 or 0.96, respectively (Fig. 5*C*).

We next examined whether knockdown of RTN3, another ER-phagy receptor, affects transport of STIM1 from the ER to autolysosomes. STIM1-EGFP-mCherry was transfected into the control siRNA, siRTN3 #1 or #2-transfected HeLa cells. Briefly, 24 h after plasmid transfection, the cells were cultured in DMEM+FBS or EBSS for 6 h. Decrease of RTN3 mRNA in the siRTN3 #1 or #2-transfected cells was confirmed by quantitative PCR (Fig. S5*A*). Both formation of EGFP^-^/mCherry^+^ puncta and PCC of EGFP and mCherry signals in the siRTN3 #1 or #2-transfected cells were comparable to those in the control siRNA-transfected cells (Fig. S5, *B* and *C*), suggesting that RTN3 knockdown does not affect transport of STIM1 from the ER to autolysosomes. Collectively, these results indicate that FAM134B knockdown specifically reduces transport of STIM1 from the ER to autolysosomes.

We next examined whether overexpression of FAM134B reverses reduction of transport of STIM1 from the ER to autolysosomes in the FAM134B knockdown cells. FLAG-EV or FLAG-siFAM134B #2-resistant FAM134B-1 was transfected in the siFAM134B #2-transfected cells along with STIM1-EGFP-mCherry. 48.4% of the siFAM134B #2 and FLAG-siFAM134B #2-resistant FAM134B-1-transfected cells formed many EGFP^-^/mCherry^+^ puncta when cultured in EBSS (Fig. S6*A*). In contrast, 24.4% of the siFAM134B #2 and FLAG-EV-transfected cells formed many EGFP^-^/mCherry^+^ puncta when cultured in EBSS. The average value of PCC of the siFAM134B #2 and FLAG-siFAM134B #2-resistant FAM134B-1-transfected cells was 0.96, which was comparable to that of the control siRNA-transfected cells (Fig. S6*B*). In contrast, the average value of PCC of the siFAM134B #2 and FLAG-EV-transfected cells was 0.98, which was significantly higher than that of the siFAM134B #2 and FLAG-siFAM134B #2-resistant FAM134B-1-transfected cells. These results indicate that overexpression of siRNA-resistant FAM134B-1 reverses reduced transport of STIM1 from the ER to autolysosomes in the FAM134B knockdown cells.

We further examined whether autophagosome formation is affected in the FAM134B knockdown cells. The control siRNA, siFAM134B #1 or siFAM134B #2 was transfected into HeLa cells. Briefly, 72 h after transfection, the cells were cultured in DMEM+FBS or EBSS in the presence of 50 nM Baf A1 for 6 h. Whole cell lysates were subjected to SDS-PAGE followed by immunoblotting with the anti-LC3 pAb, the anti-FAM134B pAb, and the anti-actin mAb. As expected, LC3-II in the control siRNA transfected cells was increased by EBSS (Fig. S7). The increase of LC3-II in the siFAM134B #1 or #2-transfected cells was comparable to that in the control siRNA-transfected cells. These results suggest that autophagosome formation is not affected by FAM134B knockdown, and support our idea that binding of STIM1 to FAM134B at the ER is important for degradation by ER-phagy.

Collectively, these results suggest that FAM134B knockdown reduces transport of STIM1 from the ER to autolysosomes.

### FAM134B knockdown accelerates G1 to S phase transition

Recent studies have shown that STIM1 knockdown delays G1 to S phase transition of cell cycle (20, 21, 22). Therefore, we reasoned that FAM134B knockdown accelerates G1 to S phase transition due to the increased protein amount of STIM1. The cell cycle-dependent expression of cyclins and cell dependent kinases (CDKs) tightly control the cell-cycle progression *via* phosphorylation of the target genes (33, 34). Especially, the protein amount of cyclin A, B1, and E is elevated in G1 to S phase transition (21, 35, 36). G1 to S phase transition is impaired by KO of cyclin A or a cyclin B inhibitor (37, 38), indicating that increasing the protein amount of cyclin A and B1 are important for G1 to S phase transition. Therefore, we examined the expression of cyclin A and B1 as the hallmarks for the cell cycle regulatory proteins that control the G1 to S phase transition.

**Figure 6:**
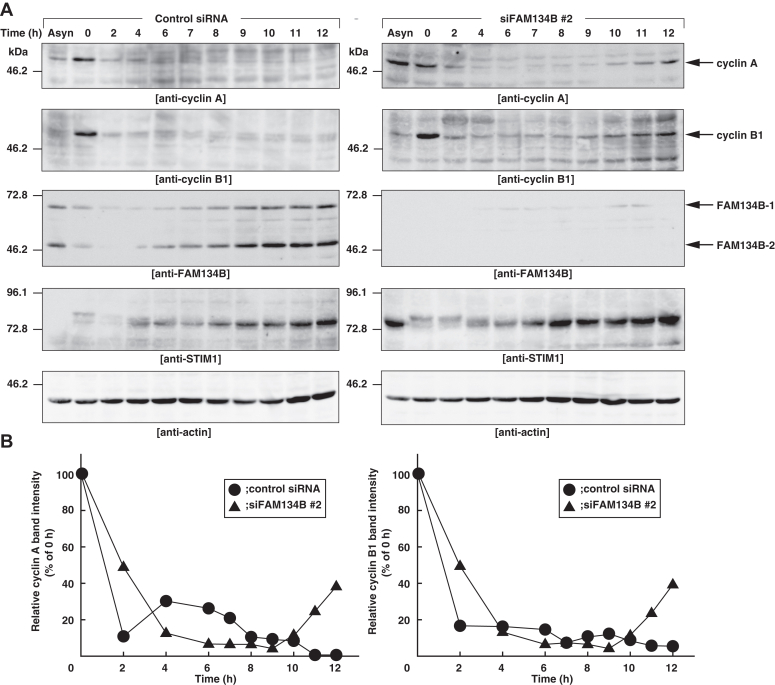
**FAM134B knockdown accelerates G1 to S phase transition.***A*, immunoblot of the FAM134B knockdown cells synchronized by thymidine and nocodazole. The control siRNA or siFAM134B #2 was transfected into HeLa cells, and the cells were cultured in the presence of 2mM thymidine for 24 h. Three hours after thymidine release, the cells were cultured in the presence of 100 ng/ml nocodazole for 12 h. Then the cells were cultured in the absence of nocodazole. The whole cell lysates of the synchronous cells that were released from nocodazole for the indicated time and the asynchronous cells (Asyn) without the thymidine-nocodazole treatment were subjected to SDS-PAGE followed by immunoblotting with the anti-cyclin A mAb, the anti-cyclin B1 mAb, the anti-STIM1 pAb, the anti-FAM134B pAb, and the anti-actin mAb. *B*, increase of cyclin A and B1 in the FAM134B knockdown cells. The intensities of immunoreactive bands for cyclin A (*left* panel) and B1 (*right* panel) in the control siRNA transfected cells (*circles*) and the siFAM134B #2-transfected cells (*triangles*) were normalized to those for actin and expressed as percentages of the cells at 0 h. FAM134B, family with sequence similarity 134 member B; STIM1, stromal interaction molecule 1.

We performed cell cycle synchronization by thymidine and nocodazole treatment to block the cells in prometaphase. The control siRNA or siFAM134B #2 was transfected into HeLa cells and the cells were cultured in the presence of thymidine for 24 h. Subsequently, 3 h after thymidine release, the cells were cultured in the presence of nocodazole for 12 h. Then the cells were cultured in the absence of nocodazole to progress mitosis. The cell lysates were subjected to SDS-PAGE followed by immunoblotting with the anti-cyclin A mAb, the anti-cyclin B1 mAb, the anti-STIM1 pAb, the anti-FAM134B pAb, and the anti-actin mAb. A previous study has shown that STIM1 is phosphorylated during mitosis (39). Therefore, molecular weight shift of STIM1 at 0 h (Fig. 6*A*) indicates that the cells were synchronized at M phase by thymidine and nocodazole treatment. In the control siRNA-transfected cells, the protein amount of cyclin A and cyclin B1 decreased as the cells progress mitosis and was kept at low levels while the cells were cultured. In contrast, in the siFAM134B #2-transfected cells, the protein amount of cyclin A and cyclin B1 increased at 10 to 12 h after nocodazole release (Fig. 6*B*), suggesting that the siFAM134B #2-transfected cells entered into G1 to S phase transition earlier than the control siRNA-transfected cells. Of note, the protein amount of FAM134B-1 and FAM134B-2 gradually increased as the cell cycles proceed and exhibited peaks at 10 to 12 h after nocodazole release. The increase of FAM134B is consistent with our idea that FAM134B transports STIM1 for degradation, thereby preventing the early entry into G1 to S phase transition.

We also examined whether FAM134B knockdown affects other periods in the cell cycle, the S and G2 phases. Cyclin B1 exhibits peaks during G2 and M phases (40). Therefore, we examined the expression of cyclin B1. The control siRNA or siFAM134B #2-transfected cells were synchronized at G1 to S phase transition using double thymidine block treatment. The cells were released from second thymidine treatment to progress S phase. The cell lysates were subjected to SDS-PAGE followed by immunoblotting with the anti-cyclin B1 mAb, the anti-FAM134B pAb, and the anti-actin mAb. Both in the control siRNA and the siFAM134B #2-transfected cells, the protein amount of cyclin B1 started to increase at 8 h after the second thymidine block, and exhibited peaks at 10 h (Fig. S8), indicating that FAM134B does not affect the duration of S and G2 phases.

Collectively, these results suggest that FAM134B knockdown accelerates G1 to S phase transition, thereby promoting cell proliferation.

## Discussion

In this study, we found that cell proliferation was promoted in the FAM134B knockdown cells. FAM134B bound to STIM1 through its C-terminal cytosolic region. The protein amount of STIM1 was increased, and transport of STIM1 to autolysosomes was reduced in the FAM134B knockdown cells. These results indicate that degradation of STIM1 through FAM134B-mediated ER-phagy is potentially involved in cell proliferation. The protein amount of FAM134B-1 and FAM134B-2 increased as the cells progressed G1 phase (Fig. 6*A*), suggesting that FAM134B-mediated ER-phagy might be required during G1 to S phase transition for protein synthesis during S phase. G1 phase is a period for cells to store enough proteins and nucleotides for DNA synthesis in the next S phase. Therefore, we speculate that a short G1 phase in the FAM134B knockdown cells would result in reducing the cell size.

FAM134B functions as an oncogene to promote tumorigenesis and metastasis in certain types of cancer such as esophageal squamous cell carcinoma and hepatocellular carcinoma by activating PI3K/akt signaling pathway (10, 41). On the other hand, FAM134B also functions as a tumor suppressor. Overexpression of a micro-RNA miR-186-5p in colon cancer cells reduces protein amount of FAM134B and promotes cell proliferation (42). In addition, higher expression of FAM134B is associated with higher survival rate of breast cancer patients (43). However, the molecular mechanism of tumor suppression regulated by FAM134B has not been understood well. Our findings that degradation of STIM1 through FAM134B-mediated ER-phagy is involved in cell proliferation might help to explain the role of FAM134B as a tumor suppressor.

In addition to FAM134B, STIM1 is both an oncogene and a tumor suppressor according to the tumor types. STIM1 overexpression promotes colorectal cancer progression, cell motility, and COX-2 expression (44). On the other hand, overexpression of STIM1 inhibits cell growth of rhabdoid tumor or rhabdomyosarcoma cell lines (45), suggesting that the protein amount of STIM1 needs to be strictly regulated for proper cell proliferation. Several reports have shown that protein synthesis of STIM1 is regulated by a set of transcription factors including microphthalmia-associated transcription factor, c-Fos, and NF-kB (46, 47). In contrast, little is known about how STIM1 protein is degraded except for the reports that STIM1 is degraded by ubiquitin-proteasome system and that mitotic STIM1 is targeted to autophagy pathway (25, 26, 27). Our findings that STIM1 is degraded through ER-phagy uncover a novel pathway of STIM1 degradation and imply that cells might use the appropriate degradation pathway according to the conditions under which they have to survive. Understanding how these two degradation pathways are used might have a potential for developing treatments for the STIM1-oriented tumorigenesis.

STIM1 is an ER Ca^2+^ sensor protein that binds to Orai1 for Ca^2+^ influx by SOCE (23, 24). FAM134B knockdown increased the protein amount of STIM1 and promoted the G1 to S phase transition (Figs. 2*A* and 6). In addition, STIM1 knockdown reversed promotion of cell proliferation of the siFAM134B #2-transfected cells (Fig. 2*C*), suggesting that increased amount of STIM1 is responsible for cell proliferation in FAM134B knockdown cells. We could not quantify the cell cycle-dependent intracellular Ca^2+^ concentration in the FAM134B knockdown cells because the quantification of Ca^2+^ concentration in G1 to S phase transition of the synchronized cells using thymidine–nocodazole block method was technically difficult. However, several studies have demonstrated that STIM1 is required for G1 to S phase transition (20, 21, 22), and that intracellular Ca^2+^ concentration is important for G1 to S phase transition (48, 49). In addition, increased amount of STIM1 promotes SOCE (50). These studies indicate that elevated Ca^2+^ concentration by STIM1-mediated SOCE promotes G1 to S phase transition. It still remains unknown how Ca^2+^ drives G1 to S phase transition. Recently, a model has been proposed by other groups that Ca^2+^-calmodulin complex phosphorylates cyclin E/CDK2 for nuclear translocation, thereby promoting transcription of genes required for G1 to S phase transition (21, 51). Combined with the model, our results suggest that increased STIM1 in the FAM134B knockdown cells elevates intracellular Ca^2+^ by SOCE, which promotes transcriptions of genes required for G1 to S phase transition. However, we cannot rule out the possibility that FAM134B knockdown proceeded G1 to S phase transition without affecting degradation of STIM1. The early entry into G1-S transition might cause the reduced degradation of STIM1 eventually in the FAM134B knockdown cells. Therefore, further biochemical analysis will be required to address whether FAM134B knockdown increases intracellular Ca^2+^.

ER-phagy is a restoration system of the damaged ER to maintain the function of the ER (17). Therefore, it has been widely accepted that ER-phagy is induced under ER stress conditions. However, several studies have demonstrated that cells undergo constitutive ER-phagy in addition to stress-induced ER-phagy (14, 15). The ER plays an essential role in intracellular Ca^2+^ homeostasis, raising a possibility that degradation of STIM1 driven by FAM134B-mediated ER-phagy might be involved in intracellular Ca^2+^ homeostasis as follows. When intracellular Ca^2+^ is elevated, CAMK2B is activated and phosphorylates FAM134B at serine 151 (12). Phosphorylated FAM134B promotes oligomerization, which generates high membrane curvature of the ER. Simultaneously, FAM134B forms a complex with STIM1, which is concentrated at the site of the ER-phagy. Then STIM1 is transported to autolysosomes through ER-phagy for degradation. Degradation of STIM1 reduces the chance of SOCE, resulting in the decrease of intracellular Ca^2+^. Further biochemical and structural studies to address whether phosphorylation of FAM134B promotes binding to STIM1 will be required.

We showed that FAM134B bound to both STIM1 and Lnp. STIM1 is degraded through FAM134B-mediated ER-phagy. In contrast, the protein amount of Lnp was not reduced by the expression of FAM134B (Fig. 3*B*), suggesting that not all the FAM134B-binding proteins are degraded through ER-phagy. It remains unclear how FAM134B recognizes its binding proteins as cargo proteins for ER-phagy. We showed that FAM134B-1 ΔC3 and ΔC4 did not bind to STIM1 (Fig. 3*C*). In contrast, Lnp bound to all the C-terminal deletion mutants (Fig. S3), suggesting that end of the C-terminal cytosolic region of FAM134B might contain a cargo recognition motif for ER-phagy. The C-terminal cytosolic region of FAM134B also comprises LIR. Therefore, biochemical analyses to examine the interaction among FAM134B, STIM1, and LC3 in a starvation-dependent manner will elucidate the molecular mechanism how ER-phagy receptors recognize cargo proteins.

Lnp preferentially localizes at the three-way junctions of the tubular ER network, thereby stabilizing the negative curvature of the three-way junctions. Lnp has two transmembrane domains which adopt a hairpin configuration, and the N-terminal cytoplasmic region of Lnp has the ubiquitin ligase activity (52, 53). Recent studies have shown that autocrine motility factor receptor ubiquitinates FAM134B and Arl6IP1 to form oligomerization, resulting in promoting ER-phagy (13, 54). FAM134B mainly localizes to the perinuclear ER sheets but it also localizes to the peripheral ER tubules (28, 54). In contrast, autocrine motility factor receptor localizes to the ER sheets (55). Therefore, Lnp might bind to and ubiquitinate FAM134B, which promotes ER-phagy at the three-way junctions of the tubular ER network.

Overall, our results underscore that FAM134B is involved in cell proliferation possibly through degradation of STIM1 through ER-phagy and imply a novel role of ER-phagy as a regulator of cell cycle.

## Experimental procedures

### siRNAs

Silencer Select siRNAs, siRNA ID: s29014 (siFAM134B #1), siRNA ID: s29013 (siFAM134B #2), siRNA ID: s13561 (siSTIM1 #1), siRNA ID: s13562 (siSTIM1 #2), siRNA ID: s20162 (siRTN3 #1), siRNA ID: s20163 (siRTN3 #2), and Silencer Select Negative Control No. 2 siRNA (Cat. No. 4390846) were purchased from Thermo Fisher Scientific.

### Plasmids

Complementary DNAs (cDNAs) encoding the human FAM134B-1, RTN3L, and SEC62 were subcloned into the pCMV vector with the N-terminal FLAG tag (pCMVFLAG). siFAM134B #2-resistant FAM134B-1 was generated by PCR and subcloned into pCMVFLAG. A series of the FAM134B-1 deletion mutants lacking 240 to 316, 317 to 359, 360 to 431, or 432 to 497 aa in the C-terminal cytosolic region (ΔC1, ΔC2, ΔC3, and ΔC4, respectively) were generated by PCR and subcloned into pCMVFLAG vector. A cDNA encoding human STIM1 was generated by PCR and subcloned into the pCMV vectors with the C-terminal mCherry and EGFP-mCherry (pCMVC-mCherry and pCMVC-EGFP-mCherry, respectively). A series of the STIM1 deletion mutants lacking 475 to 791 or 344 to 791 aa in the C-terminal cytosolic region (ΔC1 and ΔC2, respectively) were generated by PCR and subcloned into pCMVC-mCherry vector. A cDNA encoding human Lnp was subcloned into pCMVC-mCherry vector.

### Antibodies

A rabbit anti-mCherry pAb (Cat. ab183628) was purchased from Abcam. A mouse anti-actin mAb (AC-74, Cat. No. A2228) and a rabbit anti-FLAG pAb (Cat. No. F7425), were purchased from Sigma-Aldrich. A rabbit anti-LC3 pAb (Cat. PM036) was purchased from MBL. A rabbit anti-FAM134B pAb (Cat. No. 21537-1-AP) and a rabbit anti-STIM1 pAb (Cat. No. 11565-1-AP) were purchased from Proteintech. A mouse anti-cyclin A mAb (B-8, Cat. No. sc-271682) and a mouse anti-cyclin B1 mAb (GNS1, Cat. No. sc-245) were purchased from Santa Cruz Biotechnology. A rabbit anti-BiP mAb (C50B12, Cat. No. #3177) was purchased from Cell Signaling Technology.

### Cell proliferation assay

10 nM of siRNA was transfected into the cervical epithelial cancer HeLa or the prostate cancer DU145 cells with Lipofectamine RNAiMAX (Thermo Fisher Scientific) at a density of 80,000 or 40,000 cells per well in 12-well plates, respectively. Briefly, 24, 48, 72, and 96 h after transfection, the cells in the well were collected and the number of the cells were counted by hemocytometer with 0.2% trypan blue (Nacalai Tesque).

### Immunoblotting

For immunoblotting endogenous STIM1 in mammalian cells, 10 nM of the control siRNA, siFAM134B #1, siFAM134B #2, siSTIM1 #1, or siSTIM1 #2 was transfected into HeLa cells with Lipofectamine RNAiMAX, and the cells were cultured for 72 h. The whole cell lysates of the cells were subjected to SDS-PAGE followed by immunoblotting with the anti-STIM1 pAb, the anti-FAM134B pAb and the anti-actin mAb. For detecting the stress, the whole cell lysates of the cells were subjected to SDS-PAGE followed by immunoblotting with the anti-BiP pAb. For detecting autophagy, the whole cell lysates of the cells were subjected to SDS-PAGE followed by immunoblotting with the anti-LC3 pAb.

For examining the expression levels of FAM134B in mammalian cell lines, the whole cell lysates of the several mammalian cell lines were subjected to SDS-PAGE followed by immunoblotting with the anti-FAM134B pAb.

### Quantitative PCR

10 nM of the control siRNA, siFAM134B #1, or siFAM134B #2 was transfected into HeLa cells and the cells were cultured for 96 h. Total RNA was extracted from HeLa cells with RNeasy Mini kit (QIAGEN). 1 μg of total RNA was used for reverse transcription by SuperScript VILO (Thermo Fisher Scientific). Quantitative PCR was performed on LightCycler 480 Real Time PCR System (Roche) using KAPA SYBR FAST Master Mix (KAPA BIOSYSTEMS). GAPDH was used as an internal control. PCR experiments were performed in duplicate, and standard deviations were calculated and displayed as error bars.

### Coimmunoprecipitation

Appropriate combinations of plasmids were cotransfected into HEK293 cells with polyethylenimine (Polysciences), and the cells were cultured for 48 h. The cells were lysed with buffer A [50 mM Hepes-NaOH (pH 7.5), 120 mM NaCl, 1 mM EDTA, 0.1 mM ZnSO_4_, and 1% NP-40] supplemented with 10 μM 4-amidinophenylmethanesulfonyl fluoride hydrochloride, 10 μg/ml leupeptin, and 5 μg/ml aprotinin and preabsorbed with Protein G Fast Flow (GE HealthCare) at 4 °C for 15 min to remove the proteins binding to the resin nonspecifically. The supernatants were incubated with the anti-FLAG M2 affinity gel (Sigma-Aldrich) at 4 °C for 3 h. After being extensively washed with buffer A, the immunoprecipitates were subjected to SDS-PAGE followed by immunoblotting with the anti-FLAG pAb or the anti-mCherry pAb.

To check the expression of overexpressed proteins in the transfected cells, the input samples were subjected to SDS-PAGE followed by immunoblotting with the anti-FLAG pAb or the anti-mCherry pAb.

### EATR assay

An amount of 10 nM of siRNA was transfected into HeLa cells and the cells were cultured for 48 h. pCMVC-STIM1-EGFP-mCherry was transfected into the cells with Lipofectamine 2000 (Thermo Fisher Scientific), and the cells were cultured for 24 h. In case of overexpressing siFAM134B #2-resistant FAM134B-1 in the FAM134B knockdown cells, pCMVFLAG-siFAM134B #2-resistant FAM134B-1 or pCMVFLAG-EV was cotransfected with pCMVC-STIM1-EGFP-mCherry into the cells. The cells were washed twice with PBS and cultured in either DMEM+FBS or EBSS medium for 6 h.

The cells were fixed with 4% paraformaldehyde. After being washed with PBS, the cells were embedded and viewed using a microscope (Carl Zeiss, Axio Lab A1) using a 63 × oil-immersion objective lens. Collected data were exported as 8 bit TIFF files and processed using Adobe Photoshop. To statically analyze colocalization of EGFP and mCherry signals, PCC in the randomly selected cells was calculated by JACoP plugin in ImageJ (https://imagej.net/plugins/jacop) (56).

### Cell synchronization

HeLa cells were synchronized using a thymidine–nocodazole protocol or a double-thymidine protocol. Briefly, 10 nM of the control siRNA or siFAM134B #2 was transfected into HeLa cells, and the cells were cultured for 24 h. For the thymidine–nocodazole synchronization, the cells were cultured in DMEM+FBS in the presence of 2 mM thymidine (Sigma-Aldrich) for 24 h. The cells were washed twice with PBS and cultured in DMEM+FBS for 3 h. The cells were cultured in DMEM+FBS in the presence of 100 ng/ml nocodazole (Sigma-Aldrich) for 12 h. The cells were washed twice with PBS and cultured in DMEM+FBS. The whole cell lysates of the cells were subjected to SDS-PAGE followed by immunoblotting with the anti-cyclin A mAb, the anti-cyclin B1 mAb, the anti-STIM1 pAb, the anti-FAM134B pAb, and the anti-actin mAb.

For the double-thymidine synchronization, the cells were cultured in DMEM+FBS in the presence of 2 mM thymidine for 18 h. After the cells were cultured in DMEM+FBS for 9 h, the cells were cultured again in DMEM+FBS in the presence of 2 mM thymidine for 15 h. The cells were washed twice with PBS and cultured in DMEM+FBS. The whole cell lysates of the cells were subjected to SDS-PAGE followed by immunoblotting with the anti-cyclin B1 mAb, the anti-FAM134B pAb, and the anti-actin mAb.

## Data availability

All data are available in the main text or Supporting Information.

## Supporting information

This article contains supporting information.

## Conflict of interest

The authors declare that they have no conflicts of interest with the contents of this article.
