# Supplementary material for: Degradation of STIM1 through FAM134B-mediated ER-phagy is potentially involved in cell proliferation
Source: J Biol Chem. 2024 Aug 14;300(9):107674. doi: 10.1016/j.jbc.2024.107674 (PMC11414581; doi:10.1016/j.jbc.2024.107674)
Supplement: Supporting information [file mmc1.docx]

**Supporting information**

**Figure S1. HeLa cells express both FAM134B-1 and FAM134B-2, and DU145 cells express relatively high amount of FAM134B-1.**

40 µg of whole cell lysates of the cell lines as indicated were subjected to SDS-PAGE followed by immunoblotting with the anti-FAM134B pAb. The bands detected at 67 kDa and 49 kDa in HeLa cells correspond to human FAM134B-1 and FAM134B-2, respectively, as the band intensities were reduced by siFAM134B #1 or #2 (shown in **Fig. 2A**). The bands detected at 62 kDa in mouse F9 and NIH3T3 cells would correspond to mouse FAM134B-1 which is 17 amino acids shorter than human FAM134B-1. Other detected bands are assumed to be either the splicing variant form of FAM134B or non-specific signals.

**Figure S2. Accumulation of STIM1 by FAM134B knockdown does not cause ER stress.**

The control siRNA, siFAM134B #1 or siFAM134B #2 was transfected into HeLa cells. On the other hand, HeLa cells without transfection were cultured in the absence or presence of 0.3 µM thapsigargin for 8 hours (-Tg or +Tg, respectively). 15 µg of whole cell lysates were subjected to SDS-PAGE followed by immunoblotting with the anti-BiP mAb, the STIM1 pAb, the anti-FAM134B pAb and the anti-actin mAb.

**Figure S3. Lnp binds to the C-terminal deletion mutants of FAM134B.**

FLAG-EV, FAM134B-1 WT, ΔC1, ΔC2, ΔC3 or ΔC4 was co-transfected into HEK293 cells along with Lnp-mCherry. NP-40 extracts of the transfected cells were immunoprecipitated with the anti-FLAG mAb. The immunoprecipitated and the input samples were immunoblotted with the anti-mCherry pAb and the anti-FLAG pAb.

**Figure S4. FAM134B binds to the mutants of STIM1 lacking SOAR.**

The primary structures of STIM1 and the deletion mutants are represented (upper panel). TM, transmembrane. CC, coiled-coil domain. SOAR, STIM-Orai1 activating region. FLAG-EV or FAM134B-1 was co-transfected with STIM1 WT, ΔC1 or ΔC2-mCherry into HEK293 cells. NP-40 extracts of the transfected cells were immunoprecipitated with the anti-FLAG mAb. The immunoprecipitated and the input samples were immunoblotted with the anti-mCherry pAb and the anti-FLAG pAb (middle and lower panels).

**Figure S5. RTN3 knockdown does not reduce transport of STIM1 from the ER to autolysosomes.**

(A) Decrease in the amount of RTN3 mRNA in the siRTN3-transfected cells. HeLa cells were transfected with the control siRNA, siRTN3 #1 or siRTN3 #2. The amount of RTN3 mRNA in the cells was quantified by quantitative PCR. They were normalized to those of GAPDH mRNA, and expressed as percentages of the control siRNA-transfected cells. (B) No reduction of transport of STIM1-EGFP-mCherry from the ER to autolysosomes by RTN3 knockdown. HeLa cells were transfected with the control siRNA, siRTN3 #1 or siRTN3 #2, followed by transfection with STIM1-EGFP-mCherry. 24 h after transfection, the cells were cultured in DMEM+FBS or EBSS for 6 h. EGFP^-^/mCherry^+^ puncta formation in the cell was classified into three categories as described in **Fig. 4C**. Many EGFP^-^/mCherry^+^ puncta; *dark gray*, a moderate level of EGFP^-^/mCherry^+^ puncta; *light gray* and no obvious EGFP^-^/mCherry^+^ punctum; *white*. (C) PCC of EGFP and mCherry signals. Each dot indicates the value of PCC in a single cell. The averages ± SD are indicated by red lines. The data were analyzed by two-way ANOVA (DMEM+FBS/EBSS: F=247.3, *P*<0.0001; siRNAs: F=0.682, *P*=0.5084; interaction: F=6.323, P=0.0029). *****P* < 0.0001: DMEM/FBS, N. S.: not significant versus EBSS by Tukey’s *post hoc* test.

**Figure S6. Overexpression of FAM134B reverses reduction of transport of STIM1 from the ER to autolysosomes in the FAM134B knockdown cells.**

(A) Restoration of EGFP^-^/mCherry^+^ puncta formation by overexpression of FAM134B-1 in the FAM134B-1 knockdown cells. HeLa cells were transfected with the control siRNA or siFAM134B #2, followed by transfection with FLAG-EV or FLAG-siFAM134B #2 resistant FAM134B-1 along with STIM1-EGFP-mCherry. 24 h after transfection, the cells were cultured in DMEM+FBS or EBSS for 6 h. EGFP^-^/mCherry^+^ puncta formation in the cell was classified into three categories as described in **Fig. 4C**. Many EGFP^-^/mCherry^+^ puncta; *dark gray*, a moderate level of EGFP^-^/mCherry^+^ puncta; *light gray* and no obvious EGFP^-^/mCherry^+^ punctum; *white*. (B) PCC of EGFP and mCherry signals. Each dot indicates the value of PCC in a single cell. The averages ± SD are indicated by red lines. The data were analyzed by two-way ANOVA (DMEM+FBS/EBSS: F=91.24, *P*<0.0001; siRNA and FLAG-tagged protein: F=10.84, *P*<0.0001; interaction: F=9.967, P=0.0001). *****P* < 0.0001, N. S.: not significant DMEM/FBS versus EBSS, and ##*P* < 0.01, N. S.: not significant between the cells cultured in EBSS by Tukey’s *post hoc* test.

**Figure S7. FAM134B knockdown does not affect autophagosome formation.**

HeLa cells were transfected with the control siRNA, siFAM134B #1 or siFAM134B #2. 72 h after transfection, the cells were cultured in DMEM+FBS or EBSS for 6 h. 15 µg of whole cell lysates were subjected to SDS-PAGE followed by immunoblotting with the anti-LC3 pAb, the anti-FAM134B pAb and the anti-actin mAb.

**Figure S8. FAM134B knockdown does not affect G2 to M phase transition.**

The control siRNA or siFAM134B #2 was transfected into HeLa cells. 24 h after transfection, thymidine was added in the cultured medium to a final concentration of 2 mM. 18 h after thymidine treatment, the cells were cultured in the absence of thymidine for 9 h. Thymidine was added again in the cultured medium. 15 h after the second thymidine treatment, the cells were cultured in the absence of thymidine. The total cell extracts of the synchronous cells that were released from thymidine for the indicated time and the asynchronous cells without the thymidine treatment were subjected to SDS-PAGE followed by immunoblotting with the anti-cyclin B1 mAb, the anti-FAM134B pAb and the anti-actin mAb.

**Figure S9-16. Full scans of original immunoblots presented in this work.**

Figures S9A-C correspond to Figure 2A. Figures S9D-F correspond to Figure 2C. Figures S10A and S10B correspond to Figure 3A. Figures S10C and S10D correspond to Figure 3B. Figures S10E and S11A correspond to Figure 3C. Figures S11B-E and S12A-F correspond to Figure 6A. Figures S13A and S13B correspond to Figure S1. Figures S13C-F correspond to Figure S2. Figures S13G and S14A correspond to Figure S3. Figures S14B and S14C correspond to Figure S4. Figures S14D, S15A and S15B correspond to Figure S7. Figures S15C-F, S16A and S16B correspond to Figure S8. Blots were probed with antibodies indicated in the figures.
