# Supplementary material for: Degradation of STIM1 through FAM134B-mediated ER-phagy is potentially involved in cell proliferation
Source: J Biol Chem. 2024 Aug 14;300(9):107674. doi: 10.1016/j.jbc.2024.107674 (PMC11414581; doi:10.1016/j.jbc.2024.107674)
Supplement: Supporting Figures S1 to S16 [file mmc2.pdf]

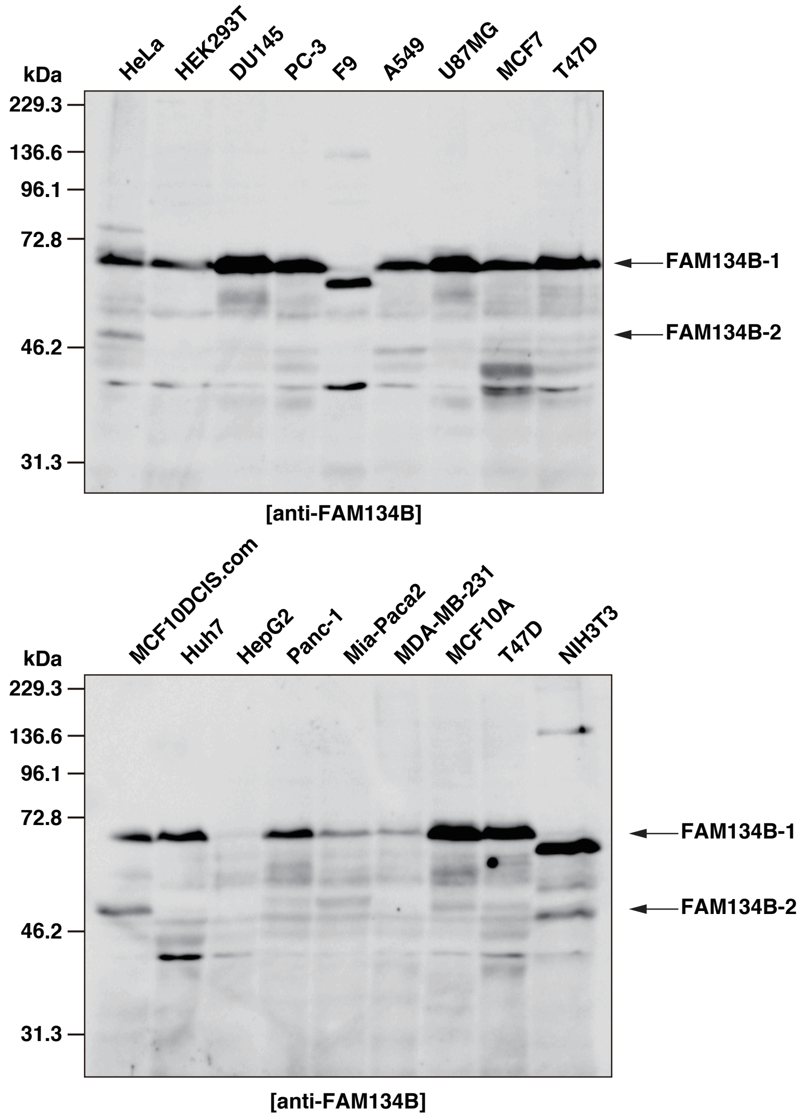
HeLa
HEK293T
DU145
PC-3
F9
A549
U87MG
MCF7
T47D
kDa
229.3
136.6
96.1
72.8
46.2
31.3
FAM134B-1
FAM134B-2
[anti-FAM134B]
MCF10DCIS.com
Huh7
HepG2
Panc-1
Mia-Paca2
MDA-MB-231
MCF10A
T47D
NIH3T3
kDa
229.3
136.6
96.1
72.8
46.2
31.3
FAM134B-1
FAM134B-2
[anti-FAM134B]

Figure S1

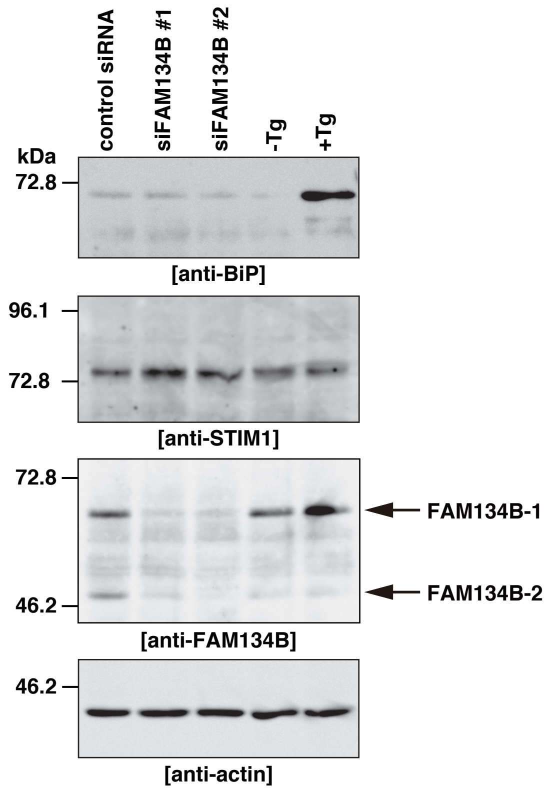
control siRNA
siFAM134B #1
siFAM134B #2
-Tg
+Tg
kDa
72.8
[anti-BiP]
96.1
72.8
[anti-STIM1]
72.8
46.2
FAM134B-1
FAM134B-2
[anti-FAM134B]
46.2
[anti-actin]

Figure S2

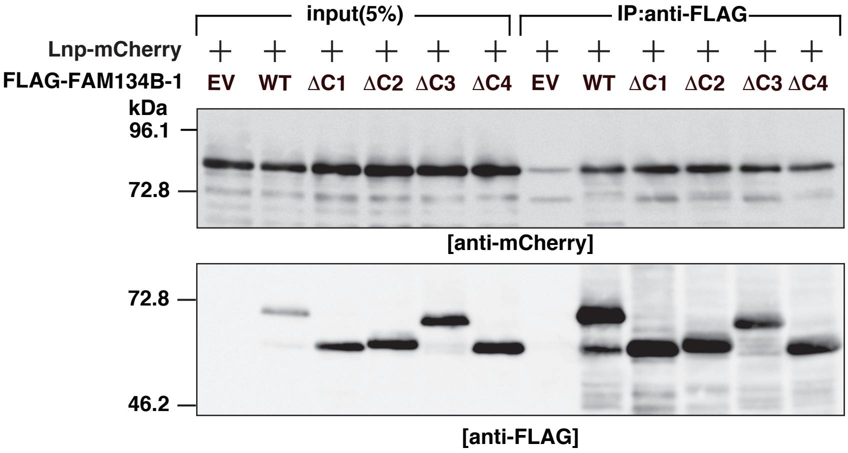
input(5%)
IP:anti-FLAG
Lnp-mCherry
+ + + + + + + + + + + +
FLAG-FAM134B-1
EV WT ΔC1 ΔC2 ΔC3 ΔC4 EV WT ΔC1 ΔC2 ΔC3 ΔC4
kDa
96.1
72.8
[anti-mCherry]
72.8
46.2
[anti-FLAG]

Figure S3

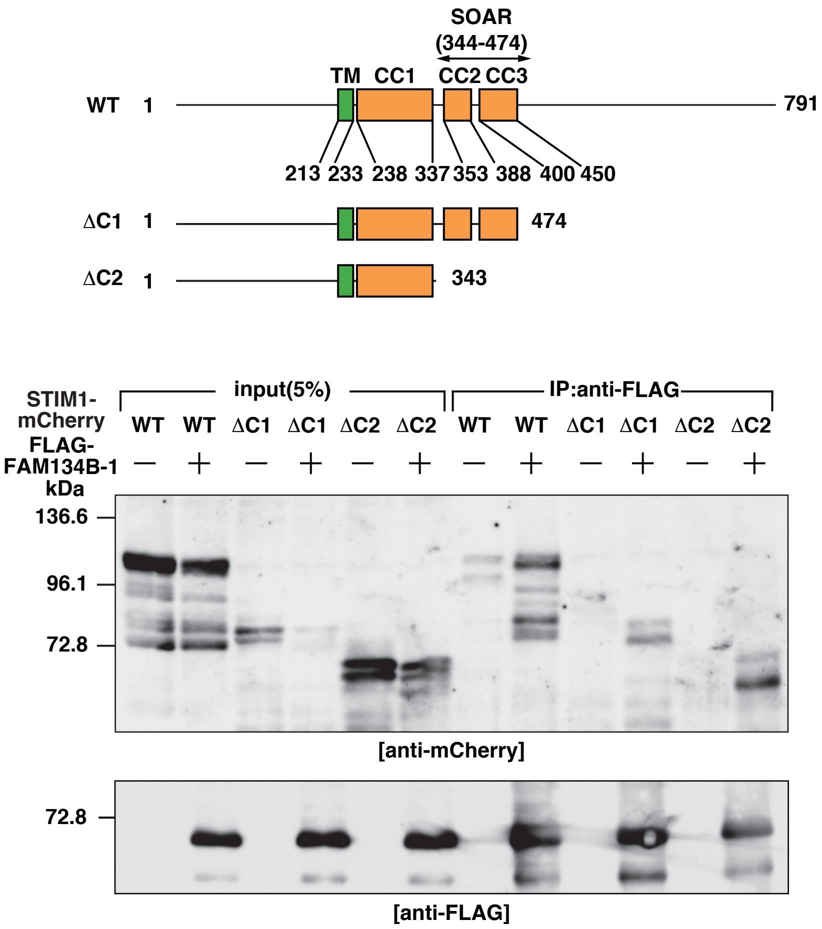
SOAR
(344-474)
TM CC1 CC2 CC3
WT 1
791
213 233 238 337 353 388 400 450
ΔC1 1
474
ΔC2 1
343
STIM1-mCherry
FLAG-FAM134B-1
kDa
input(5%)
IP:anti-FLAG
WT WT ΔC1 ΔC1 ΔC2 ΔC2 WT WT ΔC1 ΔC1 ΔC2 ΔC2
− + − + − + − + − + − +
136.6
96.1
72.8
[anti-mCherry]
72.8
[anti-FLAG]

Figure S4

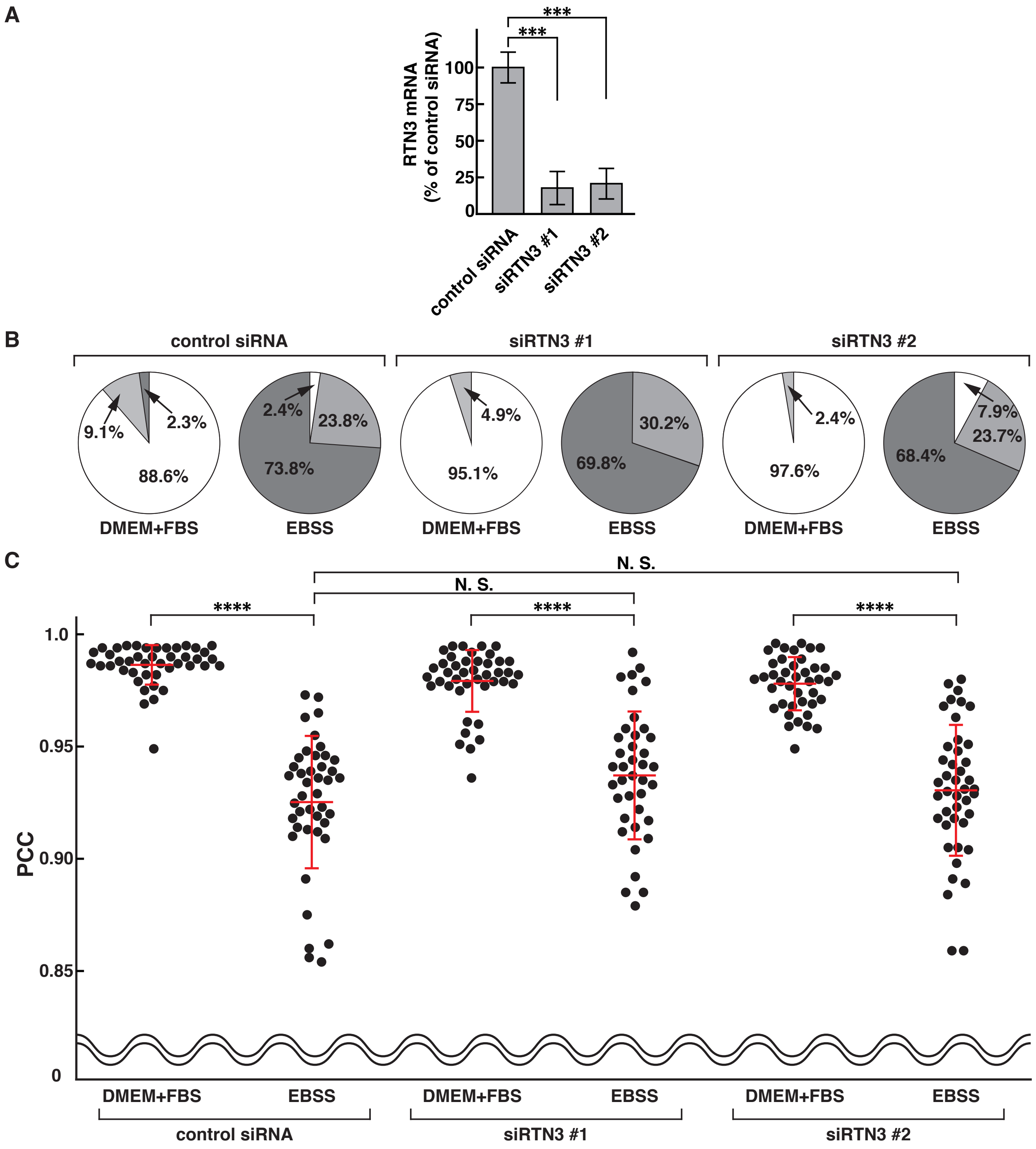
A
RTN3 mRNA
(% of control siRNA)
***
***
100
75
50
25
0
control siRNA
siRTN3 #1
siRTN3 #2
B
control siRNA
siRTN3 #1
siRTN3 #2
9.1%
2.3%
88.6%
2.4%
23.8%
73.8%
4.9%
95.1%
30.2%
69.8%
2.4%
97.6%
7.9%
23.7%
68.4%
DMEM+FBS
EBSS
DMEM+FBS
EBSS
DMEM+FBS
EBSS
C
N. S.
N. S.
****
****
****
PCC
1.0
0.95
0.90
0.85
0
DMEM+FBS
EBSS
DMEM+FBS
EBSS
DMEM+FBS
EBSS
control siRNA
siRTN3 #1
siRTN3 #2

Figure S5

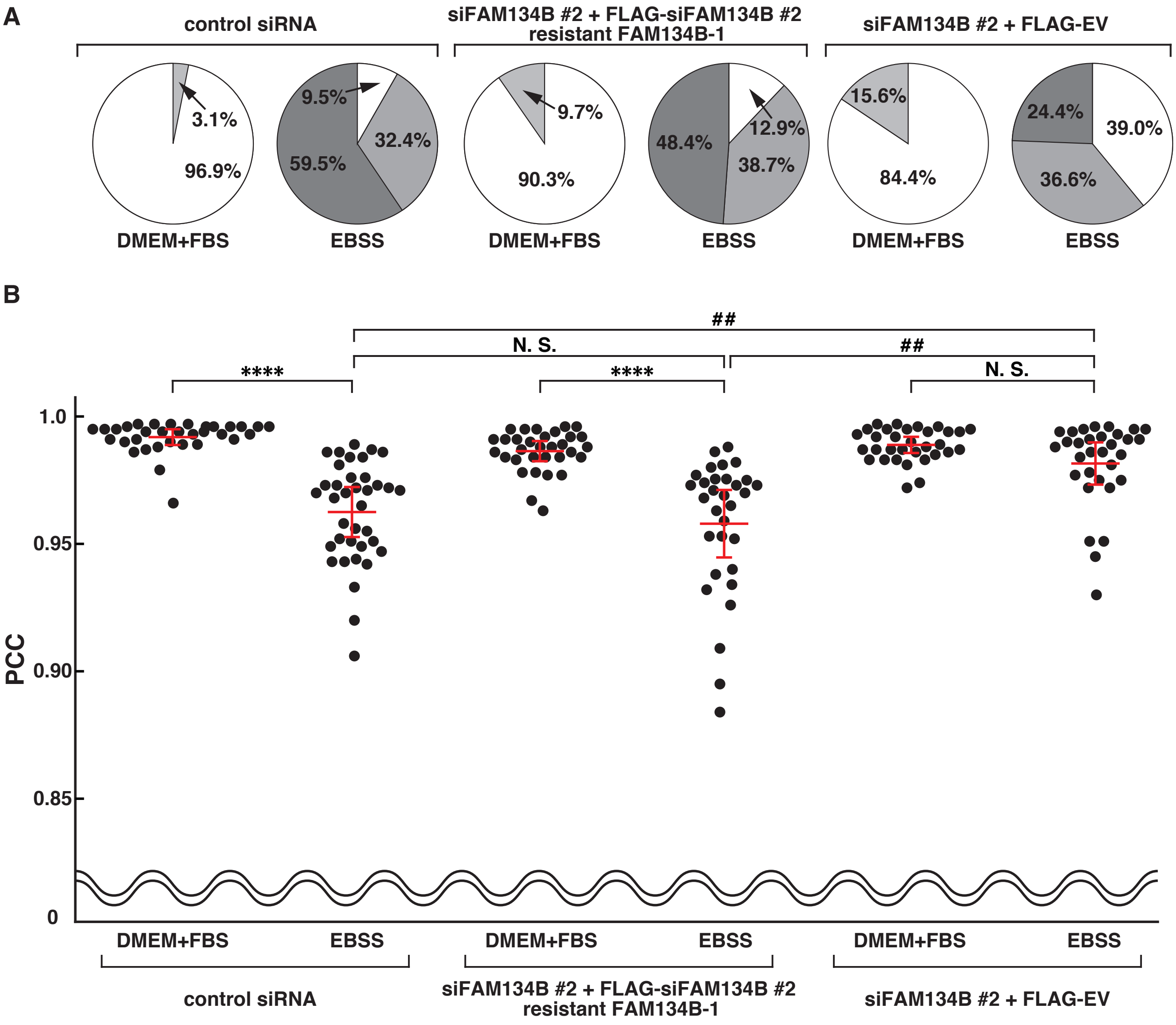
A
control siRNA
siFAM134B #2 + FLAG-siFAM134B #2 resistant FAM134B-1
siFAM134B #2 + FLAG-EV
3.1%
96.9%
9.5%
32.4%
59.5%
9.7%
90.3%
48.4%
12.9%
38.7%
15.6%
84.4%
24.4%
39.0%
36.6%
DMEM+FBS
EBSS
B
****
N. S.
##
PCC
1.0
0.95
0.90
0.85
0
control siRNA
siFAM134B #2 + FLAG-siFAM134B #2 resistant FAM134B-1
siFAM134B #2 + FLAG-EV

Figure S6

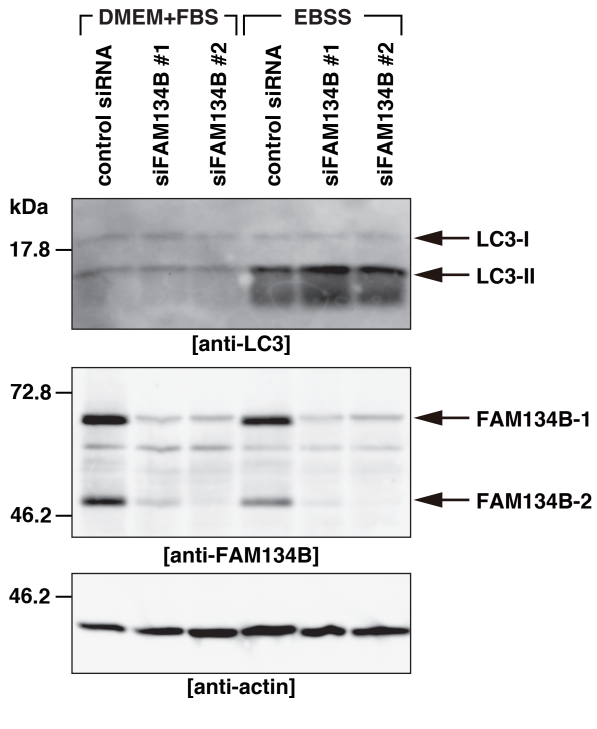

DMEM+FBS
EBSS
control siRNA
siFAM134B #1
siFAM134B #2
control siRNA
siFAM134B #1
siFAM134B #2
kDa
17.8
LC3-I
LC3-II
[anti-LC3]
72.8
46.2
FAM134B-1
FAM134B-2
[anti-FAM134B]
46.2
[anti-actin]

Figure S7

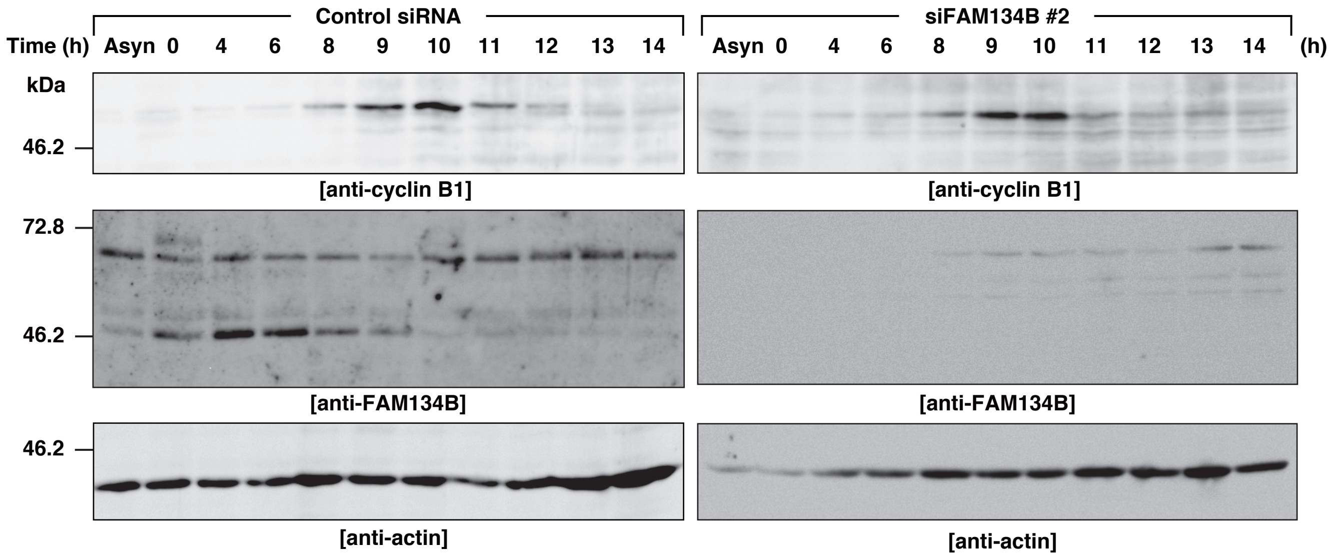
Control siRNA
siFAM134B #2
Time (h) Asyn 0 4 6 8 9 10 11 12 13 14
Asyn 0 4 6 8 9 10 11 12 13 14 (h)
kDa
46.2
[anti-cyclin B1]
[anti-cyclin B1]
72.8
46.2
[anti-FAM134B]
[anti-FAM134B]
46.2
[anti-actin]
[anti-actin]

Figure S8

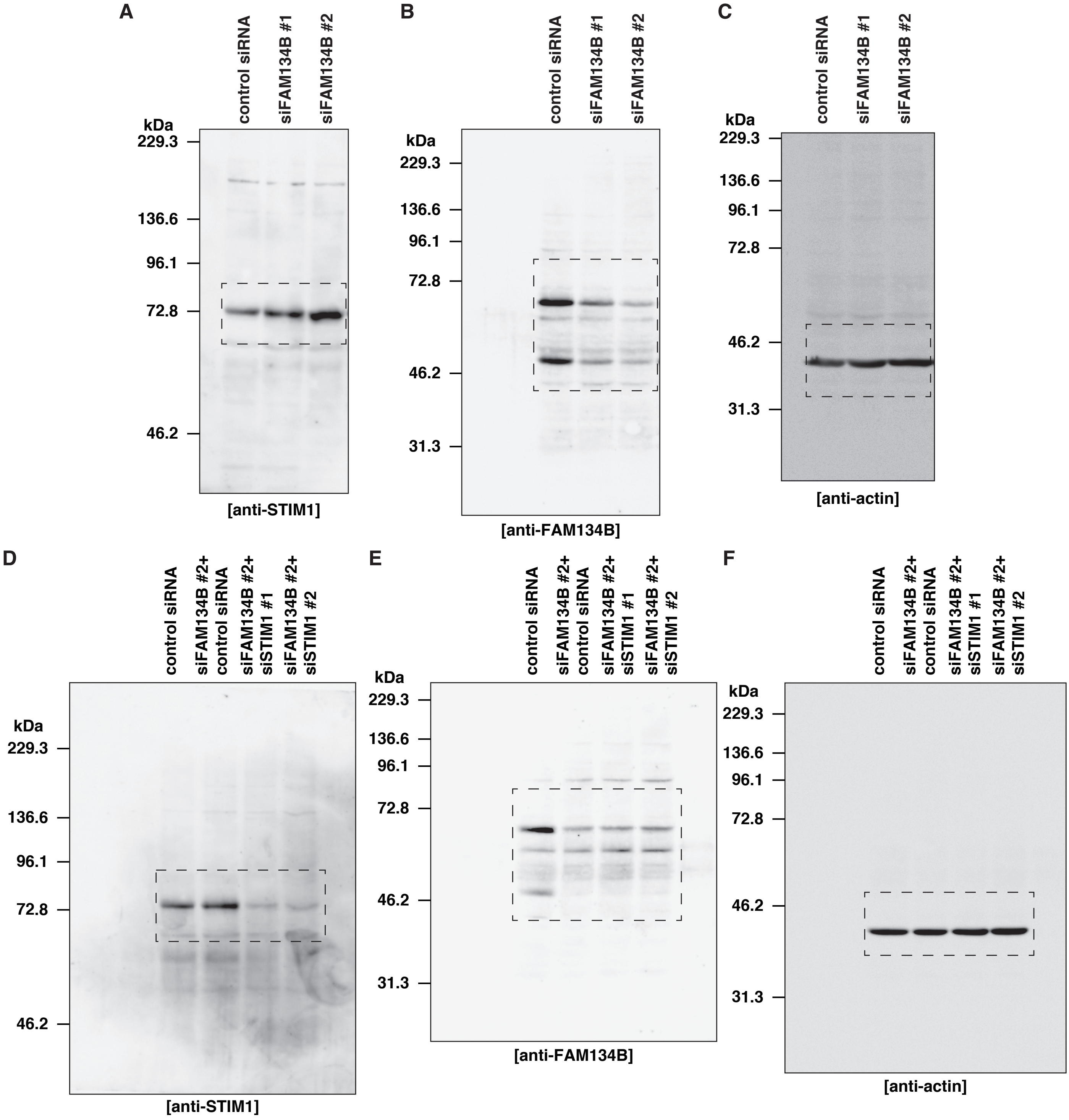
A
control siRNA
siFAM134B #1
siFAM134B #2
kDa
229.3
136.6
96.1
72.8
46.2
[anti-STIM1]
B
control siRNA
siFAM134B #1
siFAM134B #2
kDa
229.3
136.6
96.1
72.8
46.2
31.3
[anti-FAM134B]
C
control siRNA
siFAM134B #1
siFAM134B #2
kDa
229.3
136.6
96.1
72.8
46.2
31.3
[anti-actin]
D
control siRNA
siFAM134B #2+ control siRNA
siFAM134B #2+ siSTIM1 #1
siFAM134B #2+ siSTIM1 #2
kDa
229.3
136.6
96.1
72.8
46.2
[anti-STIM1]
E
control siRNA
siFAM134B #2+ control siRNA
siFAM134B #2+ siSTIM1 #1
siFAM134B #2+ siSTIM1 #2
kDa
229.3
136.6
96.1
72.8
46.2
31.3
[anti-FAM134B]
F
control siRNA
siFAM134B #2+ control siRNA
siFAM134B #2+ siSTIM1 #1
siFAM134B #2+ siSTIM1 #2
kDa
229.3
136.6
96.1
72.8
46.2
31.3
[anti-actin]

Figure S9

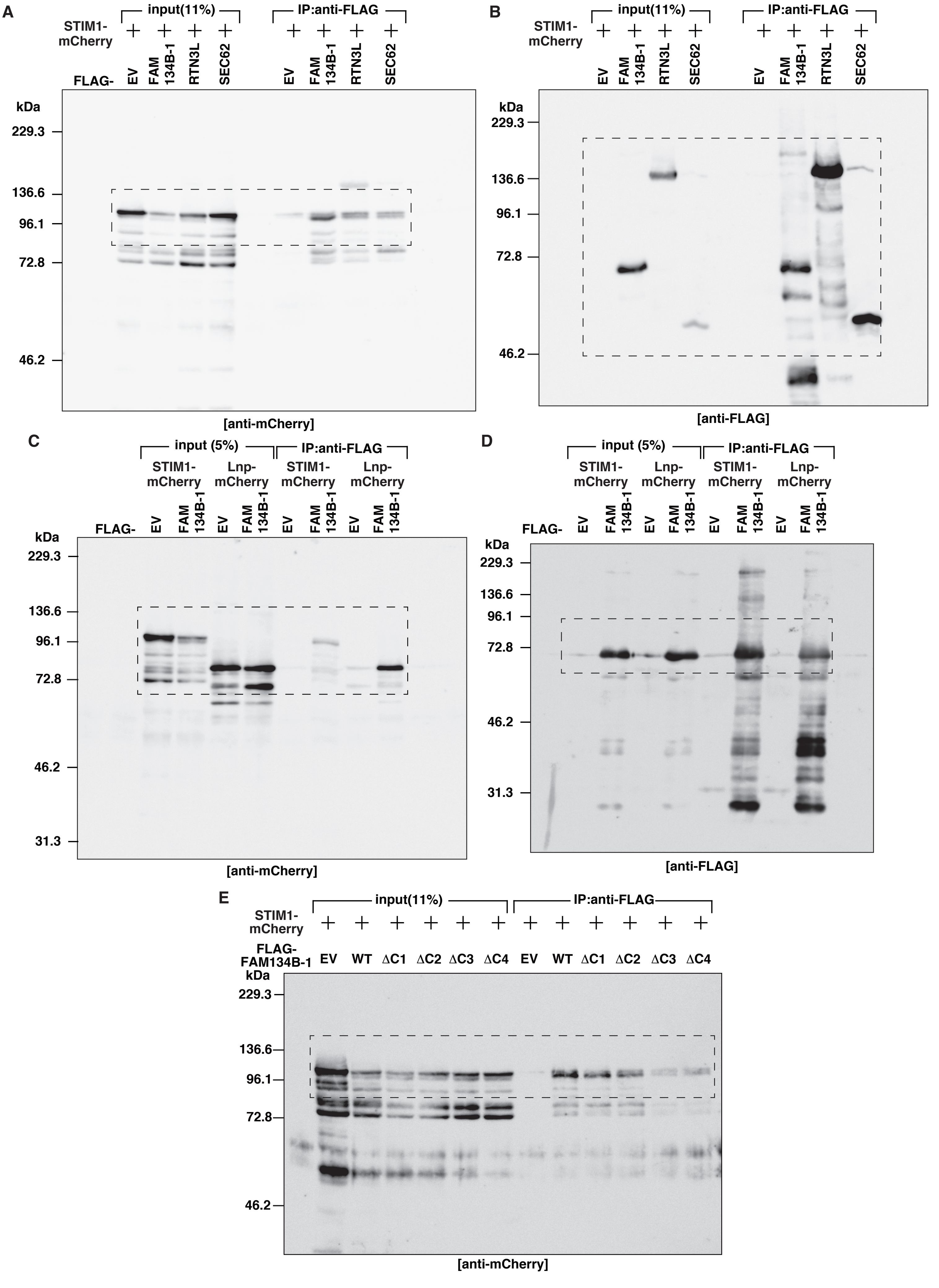
A
input(11%)
IP:anti-FLAG
STIM1-mCherry
FLAG-
EV
FAM 134B-1
RTN3L
SEC62
kDa
229.3
136.6
96.1
72.8
46.2
[anti-mCherry]
B
input(11%)
IP:anti-FLAG
STIM1-mCherry
EV
FAM 134B-1
RTN3L
SEC62
kDa
229.3
136.6
96.1
72.8
46.2
[anti-FLAG]
C
input (5%)
IP:anti-FLAG
STIM1-mCherry
Lnp-mCherry
FLAG-
EV
FAM 134B-1
kDa
229.3
136.6
96.1
72.8
46.2
31.3
[anti-mCherry]
D
input (5%)
IP:anti-FLAG
STIM1-mCherry
Lnp-mCherry
FLAG-
EV
FAM 134B-1
kDa
229.3
136.6
96.1
72.8
46.2
31.3
[anti-FLAG]
E
input(11%)
IP:anti-FLAG
STIM1-mCherry
FLAG-FAM134B-1
EV
WT
ΔC1
ΔC2
ΔC3
ΔC4
kDa
229.3
136.6
96.1
72.8
46.2
[anti-mCherry]

Figure S10

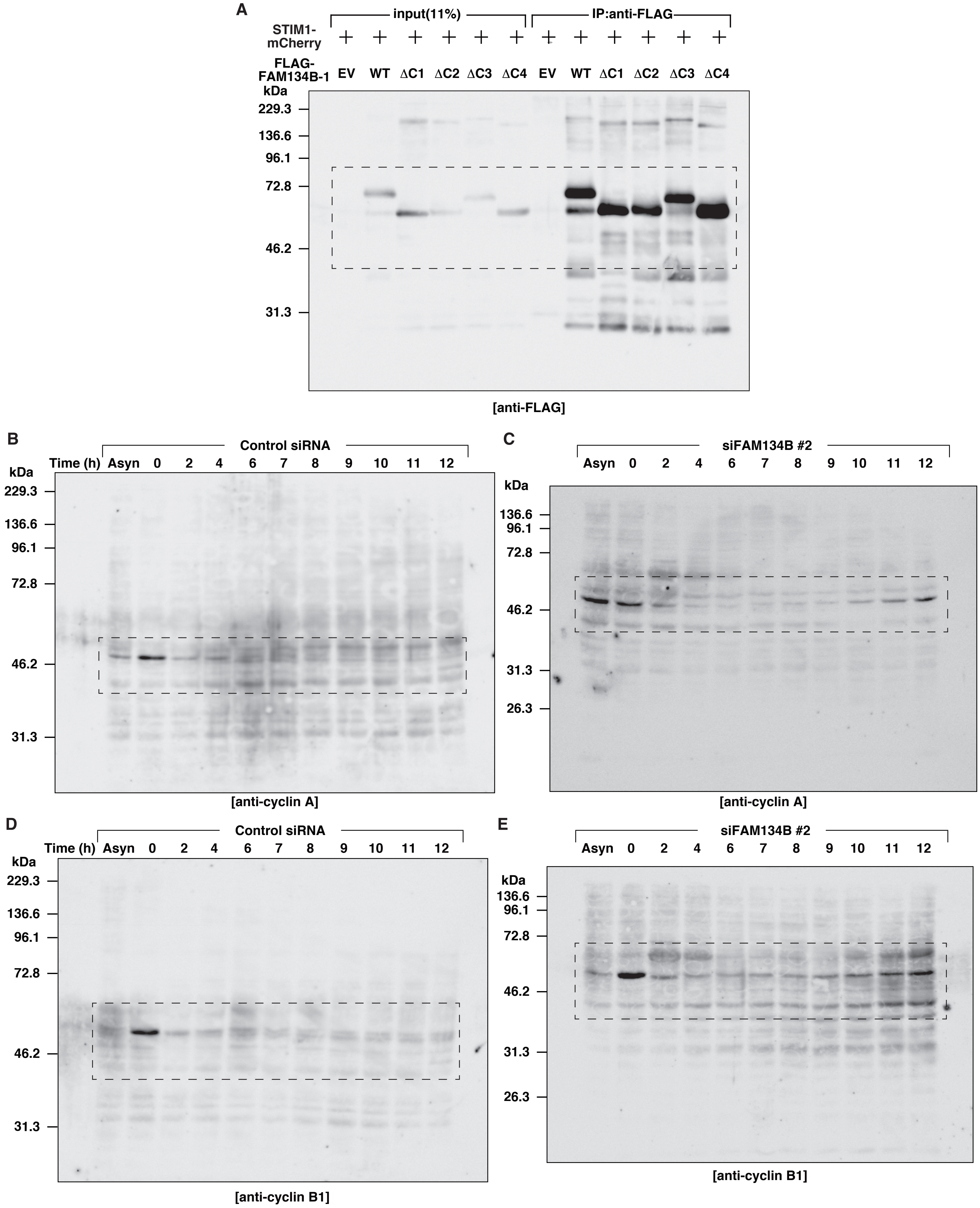
A
input(11%)
IP:anti-FLAG
STIM1-mCherry
FLAG-FAM134B-1
EV WT ΔC1 ΔC2 ΔC3 ΔC4 EV WT ΔC1 ΔC2 ΔC3 ΔC4
kDa
229.3
136.6
96.1
72.8
46.2
31.3
[anti-FLAG]
B
Control siRNA
Time (h) Asyn 0 2 4 6 7 8 9 10 11 12
[anti-cyclin A]
C
siFAM134B #2
26.3
[anti-cyclin A]
D
Control siRNA
[anti-cyclin B1]
E
siFAM134B #2
[anti-cyclin B1]

Figure S11

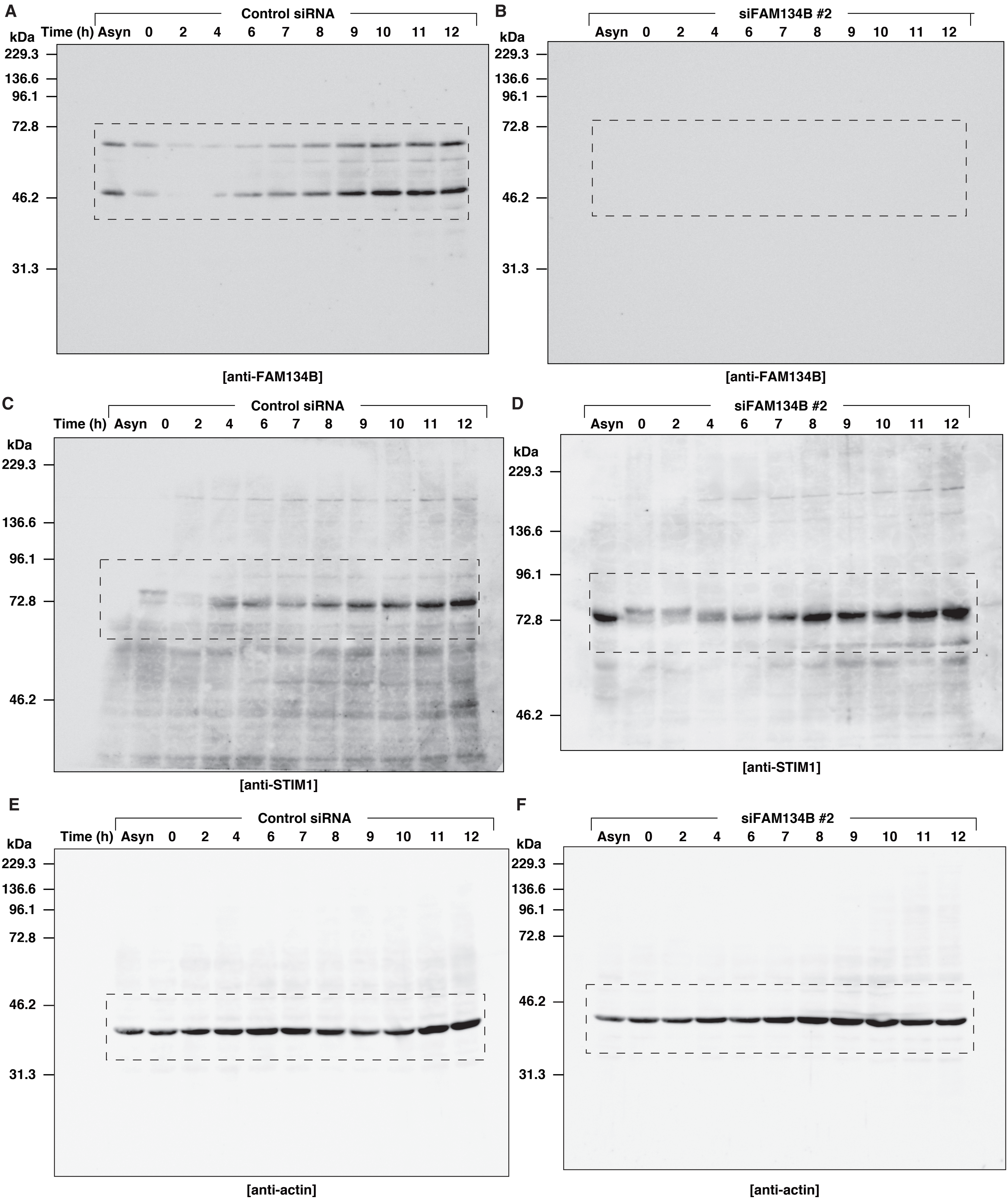
A
Control siRNA
Time (h) Asyn 0 2 4 6 7 8 9 10 11 12
kDa
229.3
136.6
96.1
72.8
46.2
31.3
[anti-FAM134B]
B
siFAM134B #2
Asyn 0 2 4 6 7 8 9 10 11 12
kDa
229.3
136.6
96.1
72.8
46.2
31.3
[anti-FAM134B]
C
Control siRNA
Time (h) Asyn 0 2 4 6 7 8 9 10 11 12
kDa
229.3
136.6
96.1
72.8
46.2
[anti-STIM1]
D
siFAM134B #2
Asyn 0 2 4 6 7 8 9 10 11 12
kDa
229.3
136.6
96.1
72.8
46.2
[anti-STIM1]
E
Control siRNA
Time (h) Asyn 0 2 4 6 7 8 9 10 11 12
kDa
229.3
136.6
96.1
72.8
46.2
31.3
[anti-actin]
F
siFAM134B #2
Asyn 0 2 4 6 7 8 9 10 11 12
kDa
229.3
136.6
96.1
72.8
46.2
31.3
[anti-actin]

Figure S12

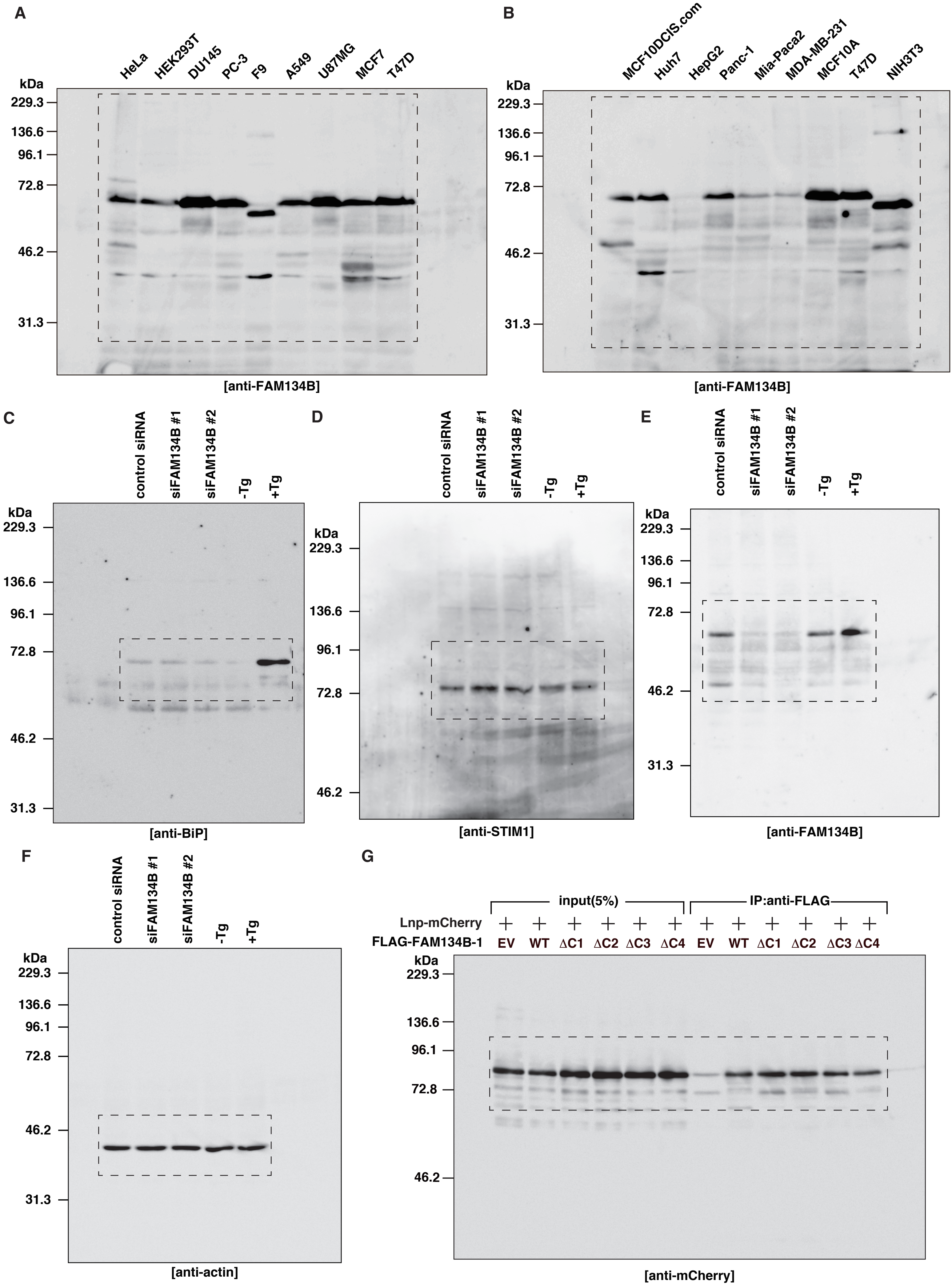
A
HeLa
HEK293T
DU145
PC-3
F9
A549
U87MG
MCF7
T47D
kDa
229.3
136.6
96.1
72.8
46.2
31.3
[anti-FAM134B]
B
MCF10DCIS.com
Huh7
HepG2
Panc-1
Mia-Paca2
MDA-MB-231
MCF10A
T47D
NIH3T3
kDa
229.3
136.6
96.1
72.8
46.2
31.3
[anti-FAM134B]
C
control siRNA
siFAM134B #1
siFAM134B #2
-Tg
+Tg
kDa
229.3
136.6
96.1
72.8
46.2
31.3
[anti-BiP]
D
control siRNA
siFAM134B #1
siFAM134B #2
-Tg
+Tg
kDa
229.3
136.6
96.1
72.8
46.2
[anti-STIM1]
E
control siRNA
siFAM134B #1
siFAM134B #2
-Tg
+Tg
kDa
229.3
136.6
96.1
72.8
46.2
31.3
[anti-FAM134B]
F
control siRNA
siFAM134B #1
siFAM134B #2
-Tg
+Tg
kDa
229.3
136.6
96.1
72.8
46.2
31.3
[anti-actin]
G
input(5%)
IP:anti-FLAG
Lnp-mCherry + + + + + + + + + + + +
FLAG-FAM134B-1 EV WT ΔC1 ΔC2 ΔC3 ΔC4 EV WT ΔC1 ΔC2 ΔC3 ΔC4
kDa
229.3
136.6
96.1
72.8
46.2
[anti-mCherry]

Figure S13

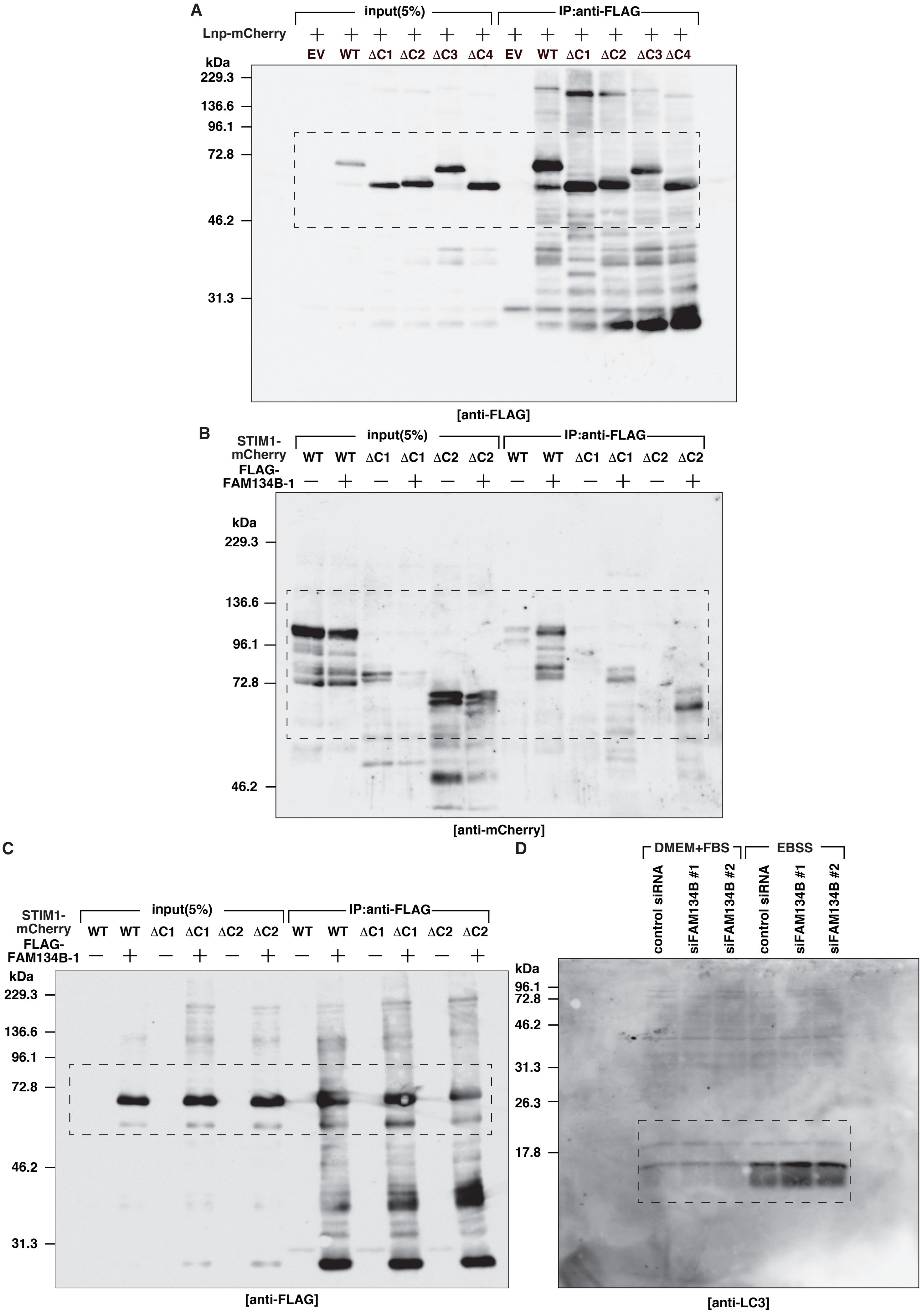
A
input(5%)
IP:anti-FLAG
Lnp-mCherry
EV WT ΔC1 ΔC2 ΔC3 ΔC4 EV WT ΔC1 ΔC2 ΔC3 ΔC4
kDa
229.3
136.6
96.1
72.8
46.2
31.3
[anti-FLAG]
B
STIM1-mCherry
FLAG-FAM134B-1
WT WT ΔC1 ΔC1 ΔC2 ΔC2 WT WT ΔC1 ΔC1 ΔC2 ΔC2
kDa
229.3
136.6
96.1
72.8
46.2
[anti-mCherry]
C
STIM1-mCherry
FLAG-FAM134B-1
input(5%)
IP:anti-FLAG
WT WT ΔC1 ΔC1 ΔC2 ΔC2 WT WT ΔC1 ΔC1 ΔC2 ΔC2
kDa
229.3
136.6
96.1
72.8
46.2
31.3
[anti-FLAG]
D
DMEM+FBS
EBSS
control siRNA
siFAM134B #1
siFAM134B #2
control siRNA
siFAM134B #1
siFAM134B #2
kDa
96.1
72.8
46.2
31.3
26.3
17.8
[anti-LC3]

Figure S14

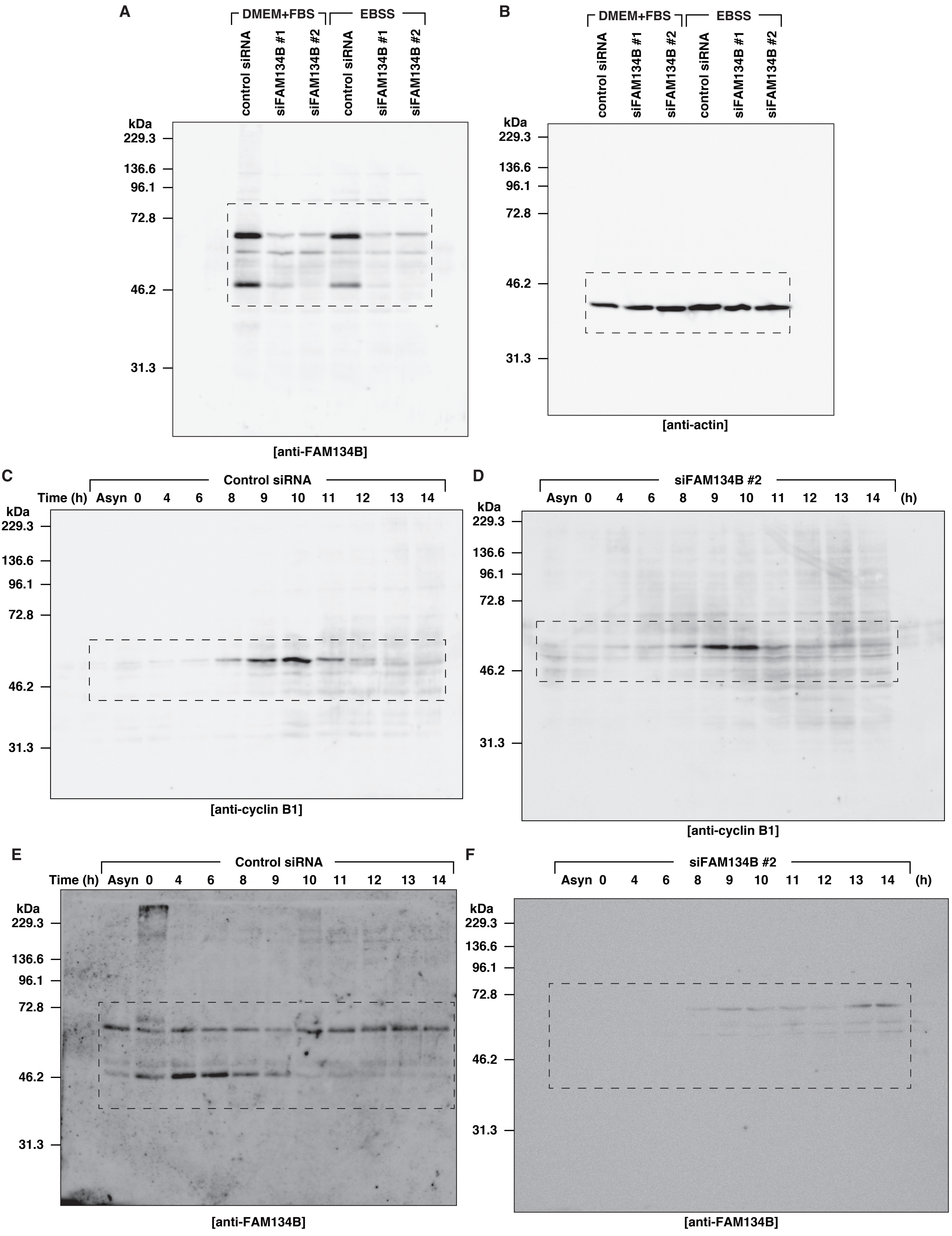
A
DMEM+FBS
EBSS
control siRNA
siFAM134B #1
siFAM134B #2
control siRNA
siFAM134B #1
siFAM134B #2
kDa
229.3
136.6
96.1
72.8
46.2
31.3
[anti-FAM134B]
B
DMEM+FBS
EBSS
control siRNA
siFAM134B #1
siFAM134B #2
control siRNA
siFAM134B #1
siFAM134B #2
kDa
229.3
136.6
96.1
72.8
46.2
31.3
[anti-actin]
C
Control siRNA
Time (h)
Asyn 0 4 6 8 9 10 11 12 13 14
kDa
229.3
136.6
96.1
72.8
46.2
31.3
[anti-cyclin B1]
D
siFAM134B #2
Asyn 0 4 6 8 9 10 11 12 13 14 (h)
kDa
229.3
136.6
96.1
72.8
46.2
31.3
[anti-cyclin B1]
E
Control siRNA
Time (h)
Asyn 0 4 6 8 9 10 11 12 13 14
kDa
229.3
136.6
96.1
72.8
46.2
31.3
[anti-FAM134B]
F
siFAM134B #2
Asyn 0 4 6 8 9 10 11 12 13 14 (h)
kDa
229.3
136.6
96.1
72.8
46.2
31.3
[anti-FAM134B]

Figure S15

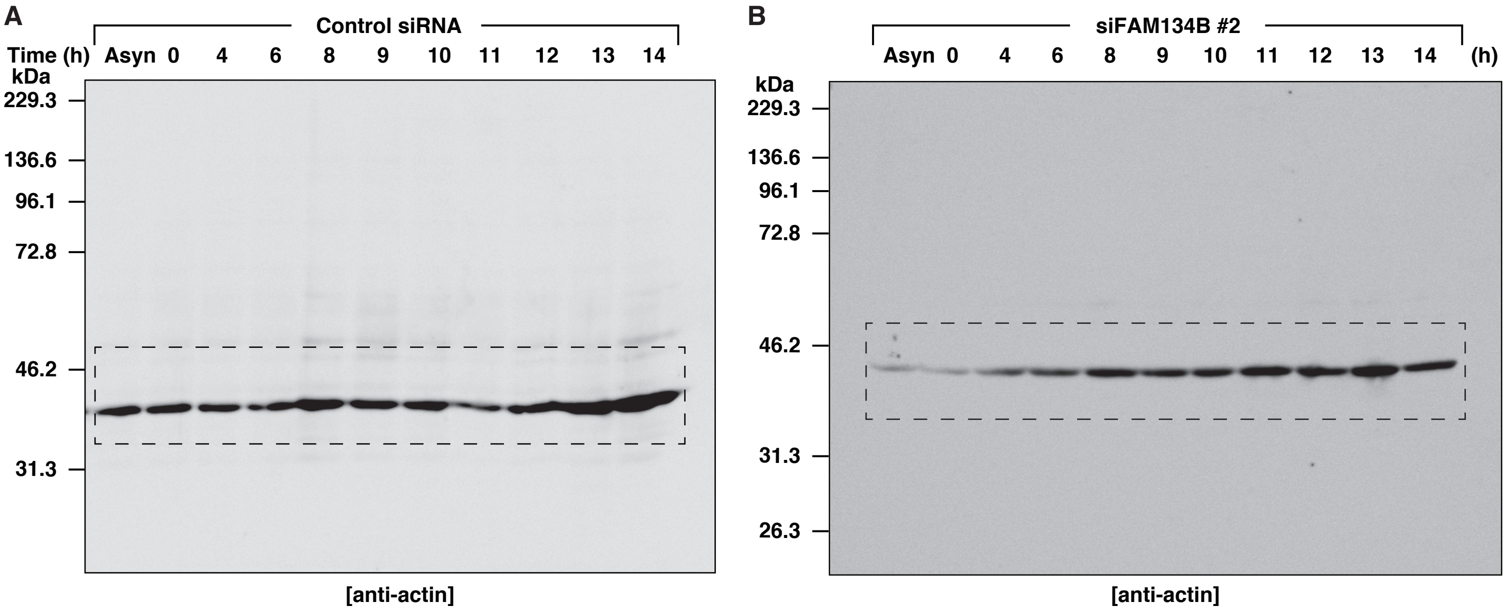
A
Control siRNA
Time (h)
Asyn 0 4 6 8 9 10 11 12 13 14
kDa
229.3
136.6
96.1
72.8
46.2
31.3
[anti-actin]
B
siFAM134B #2
Asyn 0 4 6 8 9 10 11 12 13 14 (h)
kDa
229.3
136.6
96.1
72.8
46.2
31.3
26.3
[anti-actin]

Figure S16
